# Reprogramming of lipid metabolism by ORF3a-induced microlipophagy enhances biogenesis of SARS-CoV-2 replication organelle

**DOI:** 10.1371/journal.ppat.1013676

**Published:** 2025-11-21

**Authors:** Zhifei Li, Xianfeng Hui, Miaomiao Zheng, Zhicheng He, Wenjing Huai, Binbin Ding, Meilin Jin, Yali Qin, Mingzhou Chen

**Affiliations:** 1 School of Life Sciences, Hubei University, Wuhan, China; 2 State Key Laboratory of Agricultural Microbiology, Huazhong Agricultural University, Wuhan, China; 3 State Key Laboratory of Virology and Modern Virology Research Center, College of Life Sciences, Wuhan University, LuoJia Hill, Wuhan, China; 4 Hubei Jiangxia laboratory, Wuhan, China; 5 Guangzhou Laboratory, Guangzhou, China; Tsinghua University, CHINA

## Abstract

Infection by positive-strand RNA viruses necessitates membrane expansion and elevated phospholipid biosynthesis, whereby fatty acids stored as triacylglycerols in lipid droplets (LDs) are mobilized to promote metabolic processes and membrane biogenesis. The replication organelles (ROs) of coronavirus associate with modified host endomembrane; however, the molecular mechanisms underlying the expansion and modification of these membranes remain poorly understood. Here, we show that viral protein orf3a collaborates with nsp3, nsp4, nsp6 to facilitate the formation of ROs in SARS-CoV-2. Importantly, orf3a targets LDs to ROs, establishing novel membrane contact sites and induces host cell microlipophagy, which supplies essential lipids for RO biogenesis. Subsequently, Following the formation of ROs, nsp3, with assistance from nsp12, indirectly recruits phosphatidylinositol 4-kinase beta (PI4KB) to ROs, to produce phosphatidylinositol 4-phosphate (PI4P). This action creates a PI4P-enriched microenvironment that enhances SARS-CoV-2 replication. Our findings elucidate the mechanism governing RO generation during SARS-CoV-2 infection and suggest that targeting microlipophagy pharmacologically may represent a promising strategy for the development of anti-coronaviruses therapies.

## Author summary

Viruses are masters of repurposing our cellular machinery. Positive-strand RNA viruses, including SARS-CoV-2, commandeer our cells’ membranes to build specialized compartments called replication organelles (ROs), which are essential for the virus to multiply. However, the process of how the virus constructs these ROs has been unclear. In this study, we uncover a coordinated strategy employed by SARS-CoV-2. We found that viral proteins work together to form the ROs. A key finding is that the viral protein orf3a steers cellular lipid droplets—fat storage units—towards these ROs. This connection triggers a process called microlipophagy, which breaks down the fat droplets to supply the essential building blocks (lipids) needed to expand the viral replication membranes. Furthermore, after the ROs are established, other viral proteins recruit a host enzyme, PI4KB, to generate a specific lipid signal, phosphatidylinositol 4-phosphate (PI4P). This creates a favorable microenvironment that boosts virus replication. Our work reveals a two-step “supply and enhance” mechanism for RO formation and suggests that blocking the microlipophagy step could be a promising new therapeutic strategy to combat coronavirus infections.

## Introduction

Coronaviruses (CoVs), as positive-strand RNA viruses, replicate their large genomes within the cytoplasm of host cells [1,2]. The RNA replication organelles (ROs) of CoVs are associated with a modified host endoplasm reticulum (ER) and are characterized by the presence of double-membrane vesicles (DMVs), which serve as the primary component and central hubs for viral RNA synthesis [3,4,5,6]. These DMVs are hypothesized to establish a microenvironment that facilitates RNA synthesis by enriching the localized concentration of essential metabolites, viral replicase complexes, and necessary cofactors. Moreover, these structures protect viral RNA from degradation and sensing by pattern recognition receptors of the innate immune system, thus enhancing the virus’s ability to replicate efficiently within the host [7,8].

Insights into the potential molecular mechanisms underlying the formation of ROs have emerged from transmission electron microscopy studies of cells expressing individual nonstructural proteins (nsps) or their combinations. Prior evidence indicates that nsp3, nsp4, and nsp6 of CoVs participate in a range of homotypic and heterotypic interactions that are believed to drive the formation of ROs [9,10,11]. For example, SARS-CoV nsp3 exhibits membrane disordering and proliferation capabilities, while its association with nsp4 facilitates membrane pairing. Additionally, nsp6 is known to induce membrane proliferation, resulting in the formation of perinuclear vesicles localized around the microtubule-organizing center [9]. However, the involvement of the other viral proteins in the modification and remodeling of membranes remains to be determined.

The formation of ROs is critically dependent on lipid participation, which facilitates membrane deformations and extensions necessary for achieving the appropriate topology [12]. Previous study indicated that enteroviruses can exploit various components of the cellular secretory pathway to generate organelles specialized for replication, characterized by distinct protein and lipid compositions compared to the host cell [13,14]. Notably, rhinovirus replication depends on host factors that enhance cholesterol absorption to facilitate the formation of ROs. Additionally, enteroviral protein are known to recruit LDs to ROs, establishing membrane contact sites that engage with host lipolytic machinery to transfer fatty acids from LDs, thus providing lipids essential for RO biogenesis [15]. Furthermore, both autophagy and the secretory pathways playing significant roles in this process; for instance, the silencing of the small GTPase GBF1 via siRNA was shown to reduce the number of murine hepatitis virus (MHV) ROs and hinder viral replication [16]. However, the mechanism through which SARS-CoV-2 utilizes autophagy to facilitate RO formation remain poorly understood. Recent studies suggest that lipid metabolism and pathological inflammation are perturbed in COVID-19 patients [17,18], indicating that SARS-CoV-2 may actively readjust lipid metabolism to enhance its replication capabilities.

Positive-strand RNA viruses remodel intracellular membranes to form ROs instead of directly replicating on pre-existing organelles. This remodeling is essential because ROs harbor unique lipids and proteins that distinguish them from host cell components, with phosphatidylinositol 4-kinase (PI4K) being a prominent component. PI4K comprises four isoforms: PI4KA, PI4KB, PI4K2A, and PI4K2B. Typically, PI4K is recruited to ROs through direct interactions with viral proteins or via cytokines such as GBF1/ARF1 or ACBD3 [19,20,21]. Once at the ROs, PI4K catalyzes the production of phosphatidylinositol-4-phosphate (PI4P), which binds to and induces conformational changes in the RNA-dependent RNA polymerase (RdRp), thereby modulate its enzymatic activity. Furthermore, PI4P influences the curvature of the ROs’ membranes during viral infection, generating high-curvature membrane pockets that protect viral components from host defenses [22]. In addition, PI4P is exchanged with cholesterol to maintain essential membrane properties crucial for the shape and functionality of ROs [23]. However, the specific role of PI4K and PI4P in the context of SARS-CoV-2 remains unexplored.

In the current study, we investigate the biogenesis and functional mechanism of SARS-CoV-2 ROs. Our findings demonstrate that SARS-CoV-2 ROs are formed similarly to COP-coated vesicles during the early infection stages of infection. We show that viral protein orf3a collaborates with nsp3, nsp4, and nsp6 in the formation of these ROs. Notably, orf3a plays a critical role by recruiting LDs to the ROs, thereby establishing novel membrane contact sites. Concurrently, orf3a-mediated lipophagy facilitates the transfer of fatty acids from LDs, thereby providing lipids essential for RO biogenesis. Furthermore, nsp3 recruits PI4KB to ROs, where PI4KB generates a PI4P lipid microenvironment that reinforces the structural integrity of the ROs and enhances the replication of SARS-CoV-2. In summary, our results elucidate a significant remodeling mechanism for RO formation driven by SARS-CoV-2 viral proteins and highlight how the virus manipulates host cell lipid metabolism to promote its replication.

## Results

### Recruitment of LDs to ROs generates membrane contact sites and facilitates LDs degradation in SARS-CoV-2 infected cells

**Fig 1:**
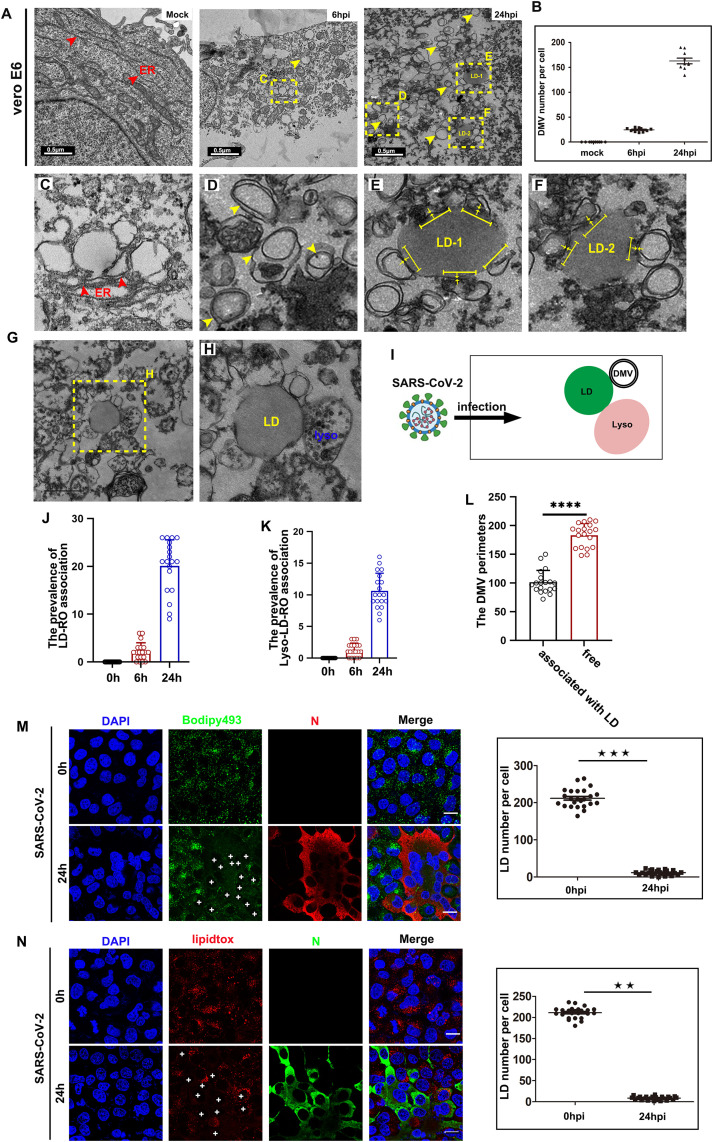
Recruitment of LDs to ROs generates membrane contact sites and facilitates LDs degradation in SARS-CoV-2 infected cells. (A) Low magnification transmission electron micrograph of SARS-CoV-2 infected VeroE6 cells with (MOI = 0.05) for 0h, 6h, and 24 h, and analyzed to access the structure of the ER and ROs. Red arrowheads indicated the ER structures, and yellow arrowheads indicated the ROs. Scale bars, 500nm.(B) Quantification of the number of ROs per cell after SARS-CoV-2 infection at 0h, 6h, 24 h. (C) Higher magnification of (A) shows that a few ROs are budding from the ER membrane in SARS-CoV-2 infected cells at 6 h. (D) Higher magnification of (A) shows heterogeneous morphology of the ROs, which shows SARS-CoV-2 remodeled the ER. The size of ROs was inconsistent. (E-F) SARS-CoV-2 induced ROs to form close membrane contacts with the LDs. Two representative images demonstrate two properties of LD-ROs membrane contacts: LD-RO contacts within a distance of 10 nm are marked by yellow arrowheads; LD surfaces within a distance of 30nm from the ROs are marked by yellow line segments. ER, endoplasmic reticulum; LD, lipid droplet. (G-H) SARS-CoV-2 induced induces the formation of a ternary complex composed of lipid droplets (LDs), lysosomes, and replication organelles (ROs). Representative image was showed. (I) The schematic of ternary complex composed of lipid droplets (LDs), lysosomes, and replication organelles (ROs) induced by SARS-CoV-2. (J-L) Quantification of the percentages of DMVs and DMV perimeters in contact with LDs, and triple-contacts between DMV-RO-lysosomes. (M) Huh7 cells were infected with SARS-COV-2 (MOI = 2) for 24 h and then analyzed to detect the number of LDs, SARS-CoV-2 was labeled with anti-N antibody (red), and LDs was labeled with Bodipy (green), and quantification of the number of LDs per cell. Scale bars, 10µm. (N) Huh7 cells were infected with SARS-COV-2 (MOI = 2) for 24 h and then analyzed to detect the number of LDs, SARS-CoV-2 was labeled with anti-N antibody (green), and LDs was labeled with lipidtox (red), and quantification of the number of LDs per cell. Scale bars, 10µm. Error bars, mean ± SD of three experiments (n = 3). Student t-test; ^★^*P* < 0.05; ^★★^*P* < 0.01; ^★★★^*P* < 0.001.

We investigated the biogenesis and functional mechanisms of SARS-CoV-2 ROs using African green monkey kidney Vero E6 cells and Hepatocellular carcinoma huh7 cells, both of which are permissive to SARS-CoV-2 infection. Initially, we analyzed the ultrastructure of SARS-CoV-2-infected Vero E6 cells. Cells were infected with SARS-CoV-2 at a multiplicity of infection (MOI) of 0.05 and chemically fixed at 0, 6, or 24 hours post-infection (hpi) for electron tomography analysis. Mock-infected cells exhibited enriched ER structures. At 6hpi, a few ROs were detectable, with their abundance increasing significantly by 24 hpi (Fig 1A and 1B). Early during infection, vesicle-like structures with indistinct bilayer membranes were observed at sites of partial endoplasmic reticulum (ER) remodeling (Fig 1C); however, these vesicles lacked clear bilayer membrane characteristics, suggesting that the initial vesicle-like structures require subsequent processing and maturation. Consistent with findings in HCV-infected cells, the membrane network is integral to RO formation, primarily deriving from the ER [24]. Notably, the bilayer membrane spacing of these vesicles was greater than that of the ER (Fig 1D), and they exhibited a remarkably uniform size of approximately 200 nm (average diameter 190.2 ± 10.5, n = 60).

Subsequently, we analyzed the ultrastructure of SARS-CoV-2-infected huh7 cells. Cells were infected at an MOI of 2 and fixed at 0, 12, or 24 hpi for electron tomography. Similar to VeroE6 cells, clusters of ROs became detectable at 12 hpi, with their abundance further increasing by 24 hpi (S1A and S1B Fig). These ROs also displayed a consistent size of approximately 205 nm (average diameter 205.9 ± 10.5, n = 60).

During the late stages of SARS-CoV-2 infection, we observed an increase in multi-membrane vesicles (MMVs) in addition to DMVs. At 24 hpi, numerous enwrapping events were evident, including ROs containing smaller ROs (S1C and S1D Fig). This extensive membrane curling and enwrapping likely contribute to the formation of MMVs, potentially driven by the accumulation of viral proteins at the ER membrane or a host autophagic response.

We also detected multiple regions of close apposition between LDs and ROs (Fig 1E and 1F). These LDs were found within 30 nm of ROs, with approximately 10%of the LD perimeter engaged in close membrane contact with the ROs. These observations align with the characteristics of membrane contact sites, defined as regions where the membranes of two organelles are within 30 nm, facilitating the transfer of signals and molecules [25]. Additionally, we identified a ternary complex comprising lysosomes, LDs and ROs, with multiple regions of close apposition between LDs and ROs, or LDs and lysosomes (Fig 1G and 1H), a schematic illustration of the ternary complex is presented in the Fig 1I. Further analysis revealed that the frequency of contact between DMV and LDs can reach approximately 20%, and the percentage of triple-contacts between DMV-RO-lysosomes was approximately 10% (Fig 1J and 1K). Moreover, statistical analysis revealed that LDs tend to approach relatively smaller DMVs, while free DMVs generally exhibit larger diameters (Fig 1L). This suggests that vesicles in contact with LDs may acquire materials, leading to further modification and maturation.

To further investigate the potential role of LDs in SARS-CoV-2 replication, we conducted time course immunofluorescence (IF) analysis to assess the distribution and abundance of LDs. LDs were labeled with the Bodipy 493 or lipidtox, which stain the core of LDs, alongside antibodies against the SARS-CoV-2 nucleocapsid protein (N). In mock-infected cells, LDs were distributed throughout the cytoplasm. In contrast, the abundance of LDs in SARS-CoV-2-infected cells decreased by 90% (Fig 1M and 1N). A consistent reduction in LDs was observed when staining for SARS-CoV-2 double-strand RNA (dsRNA) (S1E and S1F Fig). Collectively, these results indicate that, similar to other positive-strand RNA viruses, SARS-CoV-2 remodeled the ER membrane to generate DMVs as viral ROs. Following infection, LDs were recruited to lysosomes to form novel membrane contact sites, and additional membrane contact sites were established between LDs and ROs. This suggests that lipophagy may play a role in the degradation of LDs, with degraded LDs potentially transferring lipids to ROs through these membranes contact sites.

### Orf3a mediates the recruitment and degradation of LDs

**Fig 2:**
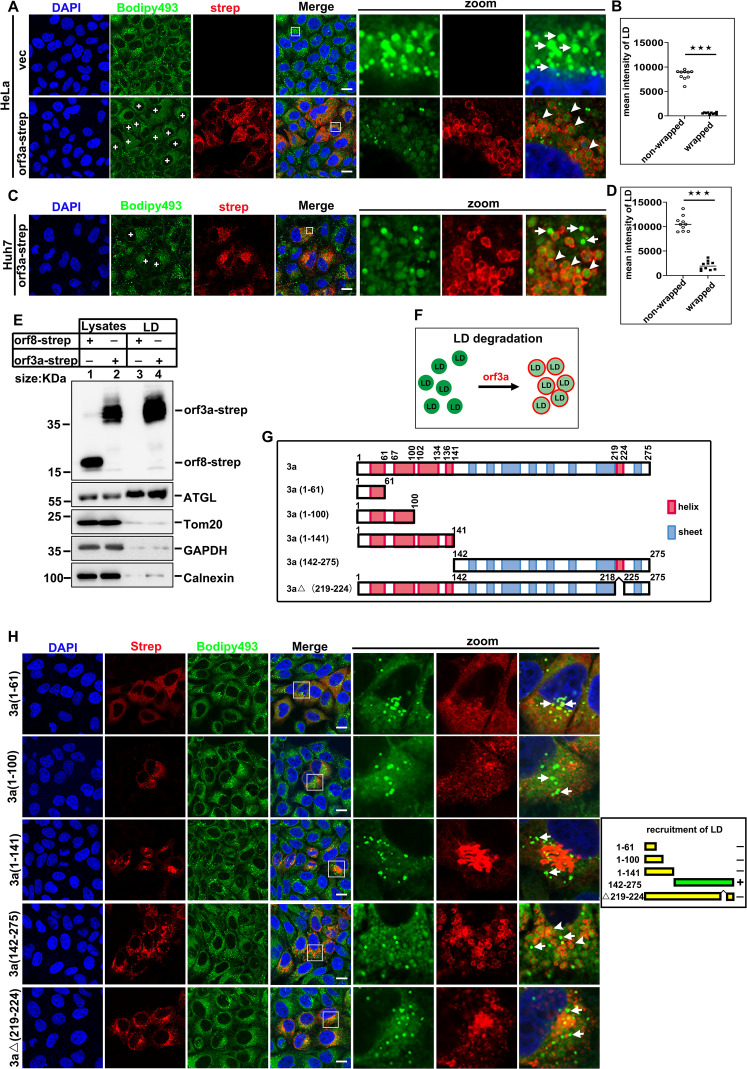
Orf3a mediates the recruitment and degradation of LDs. (A-B) Ectopically expressed SARS-CoV-2 orf3a is targeted to LDs and causes LDs degradation. (A) HeLa cells were transfected with orf3a-strep for 24 h and analyzed the distribution of LDs and orf3a. orf3a-strep was labeled with anti-strep antibodies (red), LDs were labeled with Bodipy493 (green). The plus sign marks the cells that express orf3a, the white arrows indicated the LDs which was nonwrapped by orf3a, the white arrowheads indicated the LDs which was wrapped by orf3a. (B) Quantification of the brightness of LDs which are wrapped or nonwrapped by orf3a. (C-D) Huh7 cells were transfected orf3a-strep for 24 h to confirm the distribution relationship between LDs and orf3a and quantification of the brightness of LDs wrapped or nonwrapped by orf3a. orf3a-strep was labeled with anti-strep antibodies (red), LDs were labeled with Bodipy493 (green). The plus sign marks the cells that express orf3a, the white arrows indicated the LDs which was nonwrapped by orf3a, the white arrowheads indicated the LDs which was wrapped by orf3a. (E) orf3a was localized to LDs. LD-enriched fractions were isolated from orf3a, or orf8 expressed cells at 24h and analyzed by WB. (F) The schematic of effects of orf3a on LDs. Orf3a wrapped LDs and targeting of orf3a to LDs causes the degradation of LDs. (G) Schematic of the domain structure of SARS-CoV-2 orf3a and truncation mutants in this study. The amino acids of the helix and sheet are indicated. (H) The C-terminal helix (aa219-224) of SARS-CoV-2 is essential for LD-targeting. HeLa cells were transfected with orf3a-strep or truncation mutants for 24 h and analyzed the distribution of LDs and orf3a via IF. SARS-CoV-2 orf3a-strep and truncation mutants were labeled with anti-strep antibodies (red), LDs were labeled with Bodipy493 (green). Error bars, mean ± SD of three experiments (n = 3). Student t-test; ^★^*P* < 0.05; ^★★^*P* < 0.01; ^★★★^*P* < 0.001. Scale bars, 10µm.

To gain insights into the mechanisms underlying changes in LDs distribution during SARS-CoV-2 infection, we examined the potential role of viral proteins in orchestrating these alterations. SARS-CoV-2 encodes 27 proteins with diverse functions in viral replication and assembly [26,27], which include four structural proteins: N, spike (S), envelop (E), and membrane (M) proteins. Additionally, there are 16 nonstructural proteins (nsp1–16) that are critical for viral replication, including RNA-directed RNA polymerase and helicase [28]. Furthermore, nine accessory proteins (orf3a-10) have functions in replication or packing that remain largely uncharacterized [29,30]. We employed an ectopic expression system in HeLa cells to examine the localization of individual SARS-CoV-2 protein to LDs using IF analysis. Notably, orf3a was shown to wrap around LDs, resulting in a significant decrease in the mean intensity of LDs encased by orf3a (Fig 2A and 2B). In contrast, expression of other viral proteins did not induce any observable change in LD distribution (S2A-S2C Fig). To confirm orf3a’s role in recruiting LDs and promoting their degradation, we ectopically expressed orf3a in huh7 and A549 cells, yielding consistent results across these cell types (Figs 2C and 2D, and S2D). To further confirm that orf3a target LDs, we purified LDs from cells expressing orf3a and analyzed the LD-associated proteins via immunoblotting. Orf3a was detected in the purified LD fractions, confirming IF findings. Additionally, the purified LDs contained the LD-associated enzyme adipose triglyceride lipase (ATGL) and were devoid of markers typically found in ER, mitochondria, and cytosolic compartments (Calnexin, Tom20, and GAPDH), validating the purity of these LD preparations (Fig 2E). We subsequently examined the intensity of TIP47, a protein localized to the surface of LDs. In cells transfected with empty plasmid, TIP47 exhibited a ring-like distribution around LDs. Conversely, in HeLa cells expressing strep-tagged orf3a, TIP47 displayed a homogeneous distribution throughout the cytoplasm, indicating that orf3a expression led to the displacement of TIP47 from the LD surface without affecting its overall expression level (S2E and S2F Fig). This suggests that orf3a may compete with LD membrane proteins for binding to the internal lipid core of LDs (Fig 2F).

To elucidate the mechanism by which orf3a recruits LDs, we identified the specific domains of orf3a responsible for this activity. Orf3a comprises five alpha-helices (Fig 2G). We found that truncation mutants (aa 1–61, aa 1–100, aa 1–141) lose their LD to recruit LDs, while truncation mutants (aa 142–275) remained this capability. Importantly, deletion of the the helix spanning amino acids 219–224 (∆219–224) abrogated LD recruitment (Fig 2H). These results suggested that orf3a is responsible for marking LDs for degradation, with the region encompassing amino acids 219–224 playing a critical role in this recruitment process to ROs.

### Orf3a induced-microlipophagy enhances LD degradation and free fatty acid release

We next investigate the mechanism the LD degradation. The preceding results suggest that lipophagy is implicated in the degradation of LDs. In mammalian cells, lipophagy is categorized into three distinct types: macrolipophagy, chaperone-mediated lipophagy and microlipophagy [31]. Recent evidence indicates that orf3a protein of SARS-CoV-2 inhibits autolysosome formation, thereby facilitating lysosomal exocytosis and subsequent viral egress, which in effect suppresses macrolipophagy (Chen et al., 2021; Miao et al., 2021). Chaperone-mediated lipophagy is a specific process wherein LD membrane proteins, such as perilipin (PLIN2) and perilipin 3 (PLIN3), are recognized by the cytosolic chaperone heat shock cognate protein 70 (HSC70). This interaction facilitates the delivery of LDs to lysosomes through binding with chaperone proteins. Notably, expression of orf3a did not significantly alter the distribution or protein levels PLIN3 (S3A and S3B Fig).

**Fig 3:**
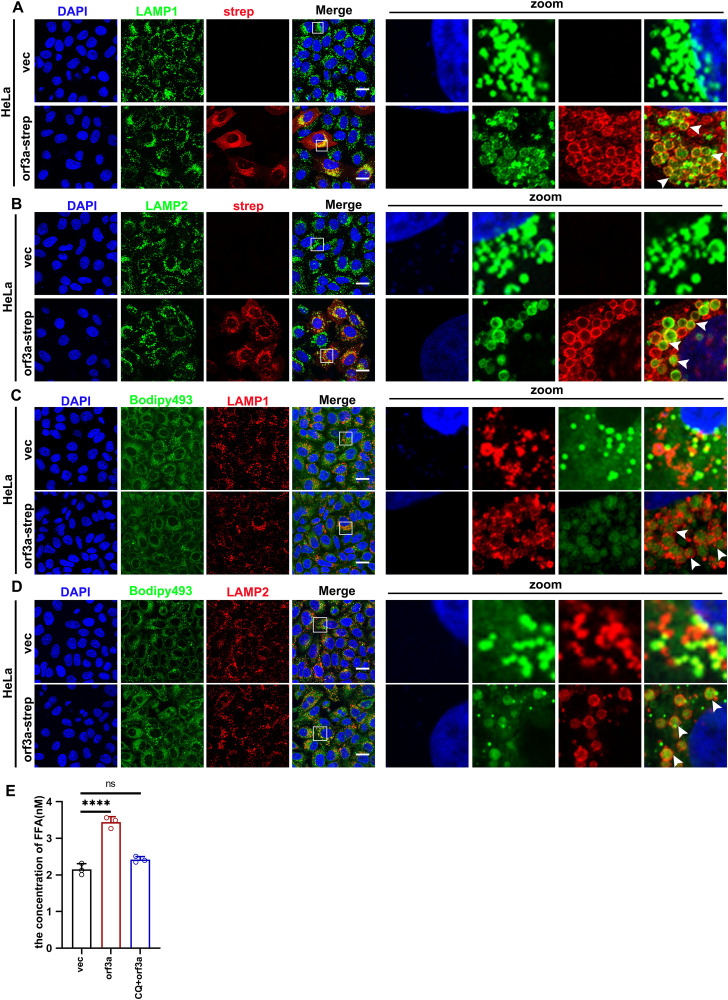
Orf3a induced-microlipophagy enhances LD degradation and free fatty acid release. (A) HeLa cells were transfected with empty plasmid or strep-tagged orf3a for 24 h and then analyzed to detect the localization of orf3a and lysosome marker LAMP1, arrowheads indicated that LAMP1 was colocalized with orf3a. (B) HeLa cells were transfected with empty plasmid or strep-tagged orf3a for 24 h and then analyzed to detect the localization of orf3a and lysosome marker LAMP2, arrowheads indicated that LAMP2 was colocalized with orf3a. (C) HeLa cells were transfected with empty plasmid or strep-tagged orf3a for 24 h and then analyzed to detect the localization of LAMP1 and LDs, arrowheads indicated that LAMP1 wrapped LDs and the wrapped LDs by LAPM1 degraded. (D) HeLa cells were transfected with empty plasmid or strep-tagged orf3a for 24 h and then analyzed to detect the localization of LAMP2 and LDs, arrowheads indicated that LAMP2 wrapped LDs and the wrapped LDs by LAPM2 degraded. (E) HeLa cells were transfected with empty plasmid or strep-tagged orf3a for 36 h, or transfected with strep-tagged and treated with CQ, then analyzed to detect the concentration of free fatty acids (FFAs) via the FFAs quantitation kit. Error bars, mean ± SD of three experiments (n = 3). Student t-test; ^★^P < 0.05; ^★★^P < 0.01; ^★★★^P < 0.001. Scale bars, 10µm.

Subsequently, we probed the possibility that orf3a induces microlipophagy. Studies indicate that lysosomes can interact with LDs to directly translocate LD components into lysosomes in the absence of autophagy-related proteins or LAMP2A chaperone [32]. We initially investigated the distribution of orf3a in relation to lysosomes. Our results showed that lysosomal markers LAMP1 and LAMP2 exhibited a ring-like distribution overlapping with that of orf3a (Fig 3A and 3B). This was corroborated in A549 cells as well (S3C and S3D Fig). Given the colocalization of LAMP proteins with orf3a, we explored the potential for these proteins to encapsulate LDs and diminish their fluorescence intensity. Our findings revealed that both LAMP1 and LAMP2 were capable of recruiting LDs; Moreover, the LDs encapsulated by LAMP1 and LAMP2 underwent degradation in cells expressing orf3a (Fig 3C and 3D), an observation consistently replicated in A549 cells (S3E and S3F Fig). Furthermore, it is known that lipophagy facilitate the breakdown of triglyerides (TG) within LDs, leading to the release of free fatty acids (FFAs). These FFAs can subsequently regenerate other lipids, promoting RO formation essential for viral replication [15]. We next determined change in FFA concentrations following orf3a expression. As anticipated, FFAs were significantly elevated in comparison to cells transfected with an empty vector (Fig 3E), providing compelling evidence that orf3a-induced microlipophagy effectively degraded TG in LDs into FFAs.

### Orf3a mediates LC3ylation of LDs

**Fig 4:**
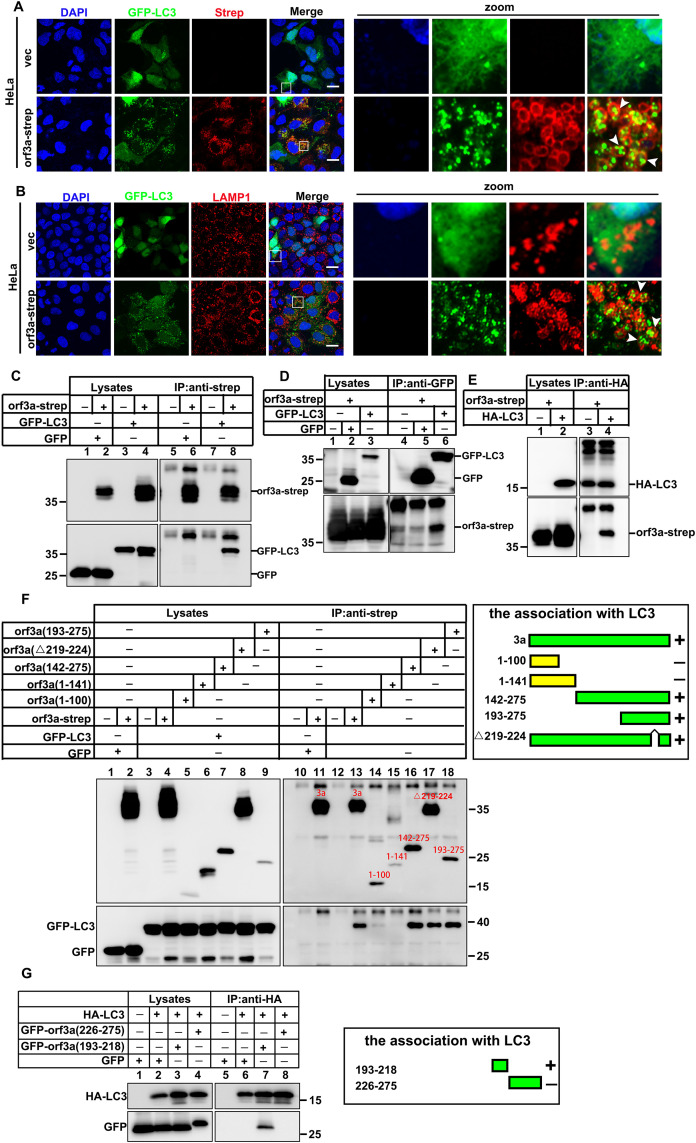
Orf3a mediates LC3ylation of LDs. (A) HeLa cells were transfected with the indicated plasmids for 24 h and then analyzed to detect GFP-LC3 localization and orf3a, arrowheads indicated autophagosomes arranged around the ring-like structures formed by orf3a. (B) HeLa cells were transfected with GFP-LC3 or GFP-LC3 and strep-tagged orf3a for 24 h and then analyzed to detect the localization of LAMP1 and GFP-LC3, arrowheads indicated that LAMP1 wrapped GFP-LC3. (C) HEK293T cells were transfected with GFP and empty plasmid, GFP and orf3a-strep, GFP-LC3 and empty plasmid, or GFP-LC3 and orf3a-strep for 36 h. Cell lysates were subjected to IP and analyzed via WB. (D) HEK293T cells were transfected with the orf3a-strep and empty plasmid, orf3a-strep and GFP, or orf3a-strep and GFP-LC3. Cell lysates were subjected to IP and analyzed via WB. (E) HEK293T cells were transfected with orf3a-strep and empty plasmid, or orf3a-strep and HA-LC3. Cell lysates were subjected to IP and analyzed via WB. (F) The C-terminal domain (193-218) or the domain (225-275) of SARS-CoV-2 orf3a is essential for LC3-association. HEK293T cells were transfected with the indicated plasmids, cell lysates were subjected to IP and analyzed via WB. (G) HEK293T cells were transfected with the indicated plasmids, cell lysates were subjected to IP and analyzed via WB. Scale bars, 10µm.

Microphagy has been shown to necessitate the conjugation of LC3 or other ATG8-family proteins, wherein ATG8ylation facilitates the delivery of specific proteins and organelles to endolysosomes, indicating its role in cargo selectivity and recognition [33,34,35]. Next, we further investigated whether orf3a present on the surface of lipid droplets (LDs) is subject to LC3ylation. HeLa cells were transfected with GFP-tagged LC3 and orf3a-strep for a duration of 24 hours. Notably, orf3a expression resulted in the formation of punctate structures labeled by GFP-LC3 in the majority of HeLa cells compared to those transfected with an empty plasmid. Remarkably, all observed LC3 puncta were localized within the ring-like structures formed by orf3a (Fig 4A). This observation corroborates that LDs also localize within the ring-like structures associated with orf3a (Fig 2A and 2C), further suggesting that LC3 puncta are recruited into these orf3a-induced structures. Subsequently, we assessed changes in the lysosomal compartment upon orf3a expression in HeLa cells. Results indicated that the lysosomal marker LAMP1 encapsulated the LC3 puncta (Figs 4B and S3G). To investigate the interaction between orf3a and LC3, we performed co-immunoprecipitation (COIP) assays. As anticipated, orf3a demonstrated a physical interaction with both GFP-LC3 and HA-LC3 (Fig 4C-4E). We further sought to delineate the critical region in orf3a that is essential for its interaction with LC3 by employing a series of orf3a deletion mutants in COIP assays. Notably, deletion of the N-terminal region encompassing residues 1–141 (orf3a142-275) did not abrogate the association between orf3a and LC3. Additionally, further deletion of the C-terminal region spanning residues 219–224 (orf3a∆219–224) maintained interaction with LC3 (Fig 4F). Further detection revealed that orf3a (193–218) directly interacted with LC3, suggesting that the C-terminal segment comprising residues 193–218 in orf3a is crucial for its interaction with LC3 and the associated LDs (Fig 4G).

### Orf3a-dependent microlipophagy enhances RO biogenesis through LD recruitment

**Fig 5:**
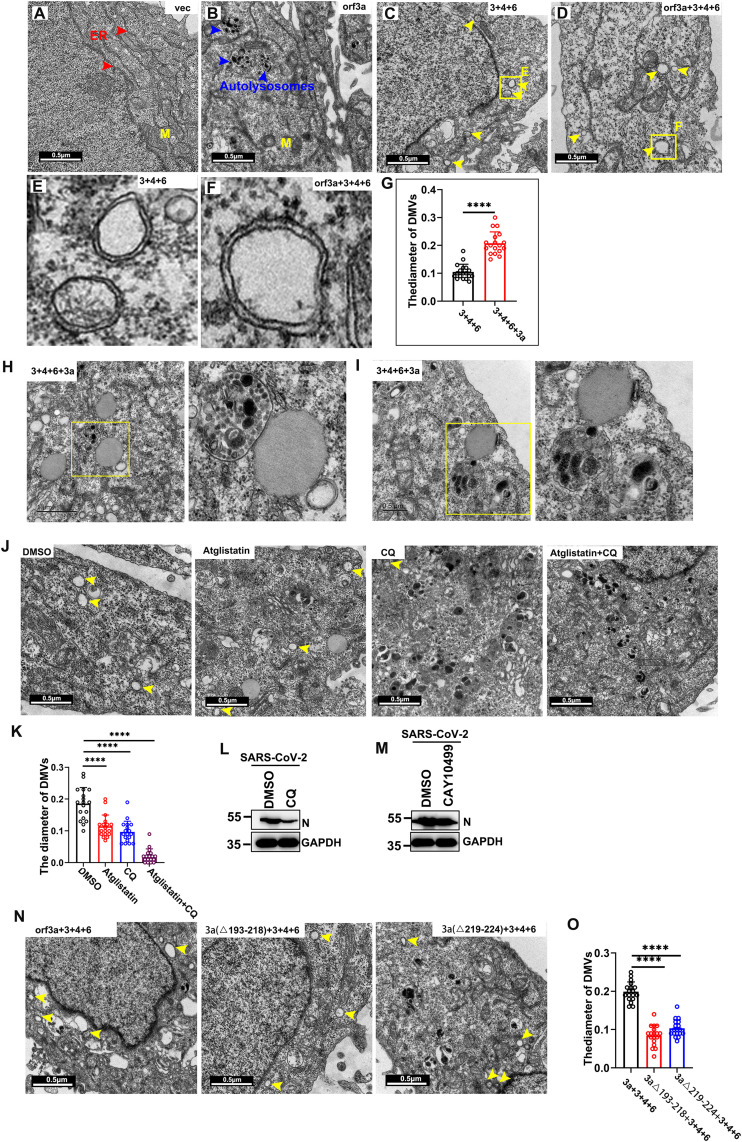
Orf3a-dependent microlipophagy enhances RO biogenesis through LD recruitment. (A-F) HeLa cells were transfected with indicated plasmids for 30 h and analyzed via electron microscopy to assess the ultrastructure of the ER and ROs. (A) ER structures were rich in HeLa cells transfected with empty plasmid. (B) Autolysosomes were found in HeLa cells transfected with Myc tagged orf3a. (C) HeLa cells were transfected with Myc-nsp3, Myc-nsp4, and Myc-nsp6 and then to assess the ultrastructure of the ROs. (D) HeLa cells were transfected with Myc-orf3a, Myc-nsp3, Myc-nsp4, and Myc-nsp6 and then to assess the ultrastructure of the ROs. (E-F) A higher magnification view of the boxed area. Red arrowheads indicate ER membrane. Blue arrowheads indicate autolysosomes. Yellow arrowheads indicate ROs. ER, endoplasmic reticulum. M, mitochondria. Scale bars, 500nm. (G) Satistical analysis on the diameter of ROs. (H-I) HeLa cells were transfected with Myc-orf3a, Myc-nsp3, Myc-nsp4, and Myc-nsp6 and then to assess the ultrastructure of the ternary complex composed of lipid droplets (LDs), lysosomes, and replication organelles (ROs). Representative image was showed. (J-K) Inhibition of microlipophagy significantly reduced the diameter of ROs. HeLa cells were transfected with Myc-orf3a, Myc-nsp3, Myc-nsp4, and Myc-nsp6 and then treated with DMSO, CQ, Atglistatin or Atglistatin and CQ for 8 h. The diameter of ROs was assessed via electron microscopy and quantified the number of ROs per cell. CQ, chloroquine, an inhibitor of lysosome degradation. Atglistatin is an inhibitor of ATGL. Yellow arrows indicate ROs. Scale bars, 500nm. (L-M) HeLa cells were infected with SARS-CoV2 and treated with CQ or CAY10499, and analyzed N protein via WB. (N-O) HeLa cells were transfected with the indicated plasmids for 36 h and analyzed the diameter of ROs via electron microscopy. Error bars, mean ± SD of three experiments (n = 3). Student t-test; ^★^*P* < 0.05; ^★★^*P* < 0.01; ^★★★^*P* < 0.001.

The aforementioned results indicate that orf3a recruits LDs to ROs and induces microlipophagy, resulting in the generation of free fatty acids (FFAs). This raises the question: is orf3a responsible for the biogenesis of ROs? To address this, we ectopically expressed either a SARS-CoV-2 empty plasmid or Myc-tagged orf3a in HeLa cells for 24 hours. Cells transfected with the empty plasmid exhibited abundant ER structures (Fig 5A). In contrast, single expression of orf3a resulted in the formation of a limited number of autolysosomes (Fig 5B), with no observable ROs present. Previous studies have identified nsp3, nsp4, and nsp6 of coronaviruses as key factors for the generation of ROs. Ectopic expression of Myc-tagged nsp3 of SARS-CoV-2 did not significantly alter cellular morphology (S4A Fig). Expression of nsp4 alone led to the formation of large areas containing single-membrane vesicles (SMVs) and maze-like bodies (MLBs) (S4B and S4C Fig). These MLBs exhibit morphologies comparable to those observed with the co-expression of SARS-CoV nsp3 and nsp4 [9]. Furthermore, electron microscopy revealed that the MLBs consisted of approximately parallel rows interspersed with double-membrane-walled circular structures, suggesting that these rows and circular structures represent longitudinal and cross-sections of closely packed double-membrane-walled tubules. Transfection of nsp6 alone also resulted in the formation of extensive areas of SMVs (S4D Fig), consistent with the observation that SARS-CoV nsp6 induces small spherical vesicles with single membranes that cluster around the microtubule organizing center [9]. Co-transfection of nsp3 and nsp4 with nsp6 resulted in pronounced alterations to membrane morphology, creating larger areas of double-membrane vesicles (DMVs) (Fig 5C). Notably, co-expression of orf3a, nsp3, nsp4, and nsp6 led to the generation of significantly larger DMVs (Fig 5C-5F) with convoluted membranes resembling those induced in SARS-CoV-2-infected cells. ROs induced by the multi-protein expression of nsp3, nsp4, and nsp6 maintained an approximate diameter of 100 nm, whereas the inclusion of orf3a resulted in ROs with an increased average diameter of 200 nm (Fig 5G). Consistent with the electron microscopy, single virus protein cannot induce significant ER morphological changes, and a multi-transfection of orf3a, nsp3, nsp4 and nsp6 made ER loose and transparent (S4F and S4G Fig). Additionally, a ternary complex comprising lysosomes, LDs, and ROs was observed upon co-expression of nsp3, nsp4, nsp6, and orf3a (Fig 5H and 5I).

In the context of microlipophagy, larger LDs experience lipolysis mediated by adipose triglyceride lipase (ATGL), resulting in a reduction of their size. Subsequently, smaller LDs establish stable membrane contact sites with lysosomes, facilitating direct lipid transfer for degradation [32]. To elucidate the role of this pathway, we employed chloroquine to disrupt the acidic environment of lysosomes and pharmacologically inhibited ATGL using atglistatin. These interventions resulted in a significant reduction in RO size (Fig 5J and 5K). Additionally, inhibitors targeting lipophagy (CQ) significantly suppressed viral replication, whereas inhibitors of lipolysis (CAY10499) had no significant impact on SARS-CoV-2 (Fig 5L and 5M).

To further confirm the essential role of orf3a-induced microlipophagy in RO generation, we co-transfected orf3a mutants with nsp3, nsp4, and nsp6. We observed a marked decrease in RO formation when utilizing the orf3a∆219–224 and orf3a∆193–218 mutants (Fig 5N and 5O), indicating that the regions of orf3a responsible for LD recruitment and LC3 association are indispensable. These findings suggest that microlipophagy induced by orf3a significantly contributes to the expansion of RO membranes.

### SARS-CoV-2 hijacks PI4KB to enrich ROs with PI4P

**Fig 6:**
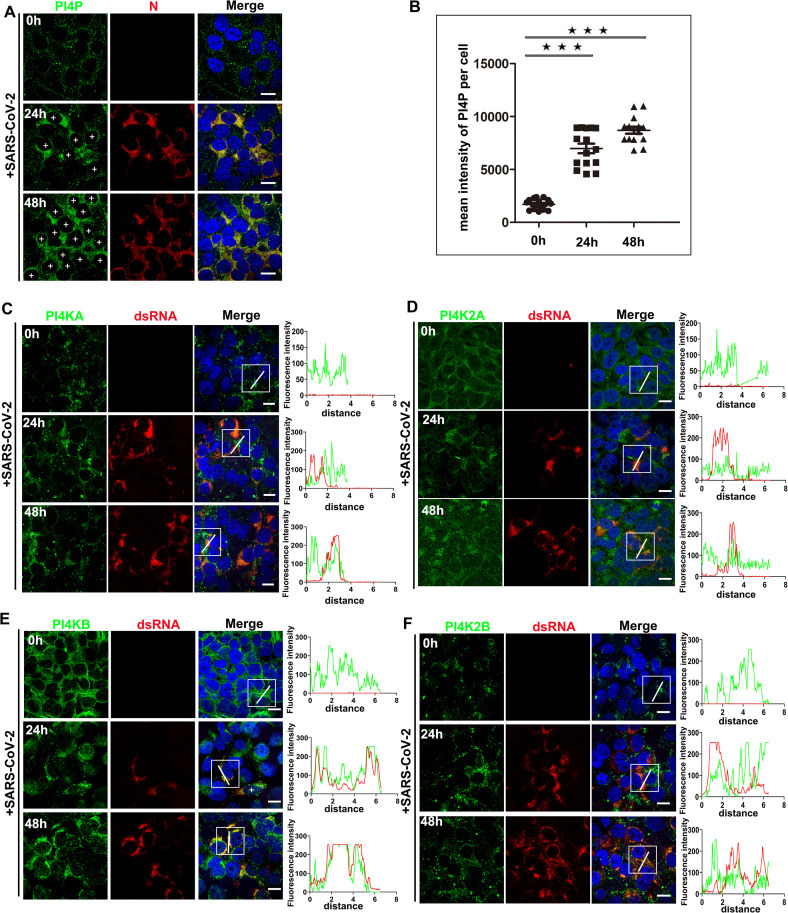
SARS-CoV-2 hijacks PI4KB to enrich ROs with PI4P. (A-B) Huh7 cells were infected with SARS-CoV-2 (MOI = 2) for consecutive times (0 h, 24 h, 48h) and then fixed and stained with PI4P (green) and N protein (red), and quantify the intensity of PI4P. The plus sign indicated the cells infected with SARS-CoV-2. (C) Huh7 cells were infected with SARS-CoV-2 (MOI = 2) for consecutive times (0 h, 24 h, 48h) and then fixed and stained with PI4KA (green) and dsRNA (red) to analyze the localization of PI4KA and dsRNA. (D) Huh7 cells were infected with SARS-CoV-2 (MOI = 2) for consecutive times (0 h, 24 h, 48h) and then analyzed to assess the localization of PI4K2A and dsRNA. (E) Huh7 cells were infected with SARS-CoV-2 (MOI = 2) for consecutive times (0 h, 24 h, 48h) and then analyzed to assess the localization of PI4KB and dsRNA. (F) Huh7 cells were infected with SARS-CoV-2 (MOI = 2) for consecutive times (0 h, 24 h, 48h) and then analyzed to assess the localization of PI4K2B and dsRNA. Error bars, mean ± SD of three experiments (n = 3). Student t-test; ^★^*P* < 0.05; ^★★^*P* < 0.01; ^★★★^*P* < 0.001. Scale bars, 10µm.

Positive-strand RNA viruses remodel the endomembrane system to form ROs instead of directly replicating on existing organelles. This remodeling is primarily driven by the unique characteristics of ROs, such as their abundant phosphatidylinositol 4-phosphate (PI4P) content, which is critical for viral replication. While LDs provide essential building blocks for RO biogenesis, additional mechanisms are necessary for the generation of PI4P to support viral replication. Therefore, we aimed to investigate whether and how SARS-CoV-2 ROs become enriched with PI4P to facilitate viral replication. Initially, we assessed the fluorescence intensity of PI4P using IF microscopy. At 24 hours post-infection with SARS-CoV-2, we observed a significant increase in PI4P fluorescence intensity in Huh7 cells compared to mock-infected controls, with intensity continuing to rise until 48 hours (Fig 6A). Quantitative analysis revealed that the average PI4P intensity in SARS-CoV-2 infected cells was approximately tenfold higher than that in mock-infected cells (Fig 6B), indicating that SARS-CoV-2 utilizes lipid synthesis pathways to replenish ROs with PI4P. PI4P is synthesized by phosphatidylinositol 4-kinases (PI4Ks), which consist of four isoforms: PI4KA, PI4KB, PI4K2A, and PI4K2B. To identify which PI4K isoform is involved in PI4P generation in SARS-CoV-2 infected cells, we examined the proximity of the four PI4K isoforms to ROs via IF. A critical step in the replication of positive-strand RNA viruses is the production of negative-stranded RNA copies of the genome, which serve as templates for genome replication by the viral RNA-dependent RNA polymerase (RdRp). Coronaviruses also generate a set of subgenomic-length negative-strand RNAs that function as templates for subgenomic mRNA synthesis [36,37]. It is widely accepted that the synthesis of viral negative-strand RNA leads to the formation of partially and/or completely double-stranded RNA (dsRNA) structures, commonly referred to as replicative intermediates, which also delineate the location of ROs.

For various positive-strand RNA viruses, the dsRNA intermediates of replication have been visualized in situ using antibodies that specifically recognize dsRNA [38,39]. In particular, monoclonal antibodies J2 [40], which recognize RNA duplexes larger than 40 base pairs, were employed in our IF assays, resulting in highly specific labeling of SARS-CoV-2 infected cells, while mock-infected cells exhibited negligible signal. Concurrently, we found that PI4KA, PI4K2A, and PI4K2B did not colocalize with dsRNA (Fig 6C and 6D and 6F), suggesting that these isoforms were not recruited to ROs for PI4P generation. In contrast, only PI4KB was found to be recruited to ROs and colocalized with dsRNA (Fig 6E). Furthermore, we observed that knockdown of PI4KB expression via shRNA significantly decreased SARS-CoV-2 replication (S5B Fig), indicating that PI4KB plays a crucial role in generating PI4P on ROs and is necessary for SARS-CoV-2 replication. Conversely, other PI4K isoforms were not required for viral replication (S5A and S5C and S5D Fig). Collectively, these results demonstrate that SARS-CoV-2 recruits PI4KB to ROs to generate PI4P, thereby enriching ROs with PI4P and promoting viral replication.

### Recruitment of PI4KB to ROs mediated by nsp12 and nsp3 interactions

**Fig 7:**
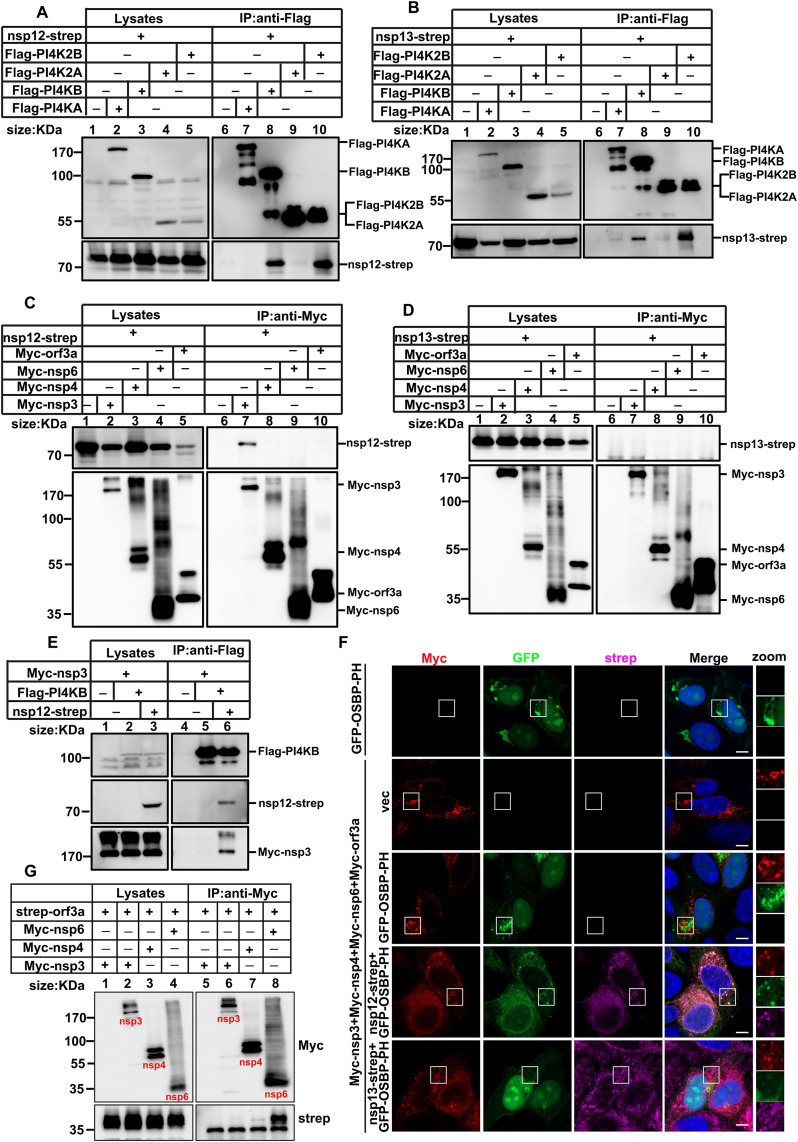
PI4KB is recruited to SARS-CoV-2 ROs via its interaction with nsp3. (A) HEK293T cells were transfected with nsp12-strep and empty plasmid, nsp12-strep and Flag-PI4KA, nsp12-strep and Flag-PI4KB, nsp12-strep and Flag-PI4K2A or nsp12-strep and Flag-PI4K2B for 36 h. Cell lysates were subjected to IP and analyzed via WB. (B) HEK293T cells were transfected with nsp13-strep and empty plasmid, nsp13-strep and Flag-PI4KA, nsp13-strep and Flag-PI4KB, nsp13-strep and Flag-PI4K2A or nsp13-strep and Flag-PI4K2B for 36 h. Cell lysates were subjected to IP and analyzed via WB. (C) HEK293T cells were transfected with nsp12-strep and empty plasmid, nsp12-strep and Myc-orf3a, nsp12-strep and Myc-nsp3, nsp12-strep and Myc-nsp4 or nsp12-strep and Myc-nsp6 for 36 h. Cell lysates were subjected to IP and analyzed via WB. (D) HEK293T cells were transfected with nsp13-strep and empty plasmid, nsp13-strep and Myc-orf3a, nsp13-strep and Myc-nsp3, nsp13-strep and Myc-nsp4 or nsp13-strep and Myc-nsp6 for 36 h. Cell lysates were subjected to IP and analyzed via WB. (E) HEK293T cells were transfected with Myc-nsp3 and empty plasmid, Myc-nsp3, Flag-PI4KB, or Myc-nsp3, nsp12-strep, and Flag-PI4KB for 36 h. Cell lysates were subjected to IP and analyzed via WB. (F) HeLa cells were transfected with the indicated plasmids and analyzed the distribution of the proteins. Scale bars, 5µm. (G) HEK293T cells ere transfected with the indicated plasmids and analyzed via WB.

To elucidate the recruitment mechanism of PI4KB to ROs, we drew on insights from previous studies demonstrating that picornaviruses employ viral proteins, particularly 3A, to facilitate this recruitment, with 3A exhibiting colocalization with ROs [19,41]. SARS-CoV-2 encodes 27 proteins, each serving diverse roles in viral replication and packaging. To explore the mechanisms underlying PI4KB recruitment during SARS-CoV-2 infection, we examined the potential roles of viral proteins in orchestrating these processes. We conducted COIP assays to assess the interactions between viral proteins and PI4Ks. As illustrated in Fig 7A and 7B, we observed that both nsp12 and nsp13 interacted with PI4KB and PI4K2B. Notably, PI4K2B did not localize to ROs during SARS-CoV-2 infection and silencing of PI4K2B via shRNA did not impact viral replication (Figs 6F and S5D). In addition, we found that all other viral proteins did not interact with PI4Ks (S6 and S7 Figs), and these results indicated that nsp12 and nsp13 primarily serve to recruit PI4KB to ROs. Since nsp12 and nsp13 do not directly participate in RO formation, their role appears to act as a bridge connecting essential RO-forming proteins to PI4KB. To verify this hypothesis, we assessed the association of nsp12 and nsp13 with other viral proteins, including orf3a, nsp3, nsp4, and nsp6. Our analysis revealed that only nsp12 interacted with nsp3 (Fig 7C), while nsp13 displayed no interactions with any RO-forming proteins (Fig 7D); Furthermore, when PI4KB was co-expressed with nsp3, there was no significant interaction detected between these proteins. However, co-expression of PI4KB with both nsp3 and nsp12 resulted in a strong interaction between nsp3 and PI4KB (Fig 7E). Subsequently, we constructed a GFP-fused OSBP PH motif. Since the OSBP-PH motif specifically binds to the product of PI4K (PI4P), it can be used to visualize the localization of PI4K. In HeLa cells co-expressing nsp3/nsp4/nsp6/orf3, punctate aggregates, ROs, appeared intracellularly. Upon further expression of nsp12, PI4P was recruited to these ROs, whereas nsp13 failed to do. This results further confirmed that nsp12 is capable of recruiting PI4K to ROs. Collectively, these findings suggest that PI4KB is recruited to ROs via a complex involving nsp3 and nsp12, thereby facilitating the localization of PI4KB essential for viral replication.

In addition, through co-immunoprecipitation experiments, we demonstrated that orf3a interacts with NSP6, suggesting that orf3a is recruited to ROs through its interaction with NSP6 (Fig 7G).

## Discussion

**Fig 8:**
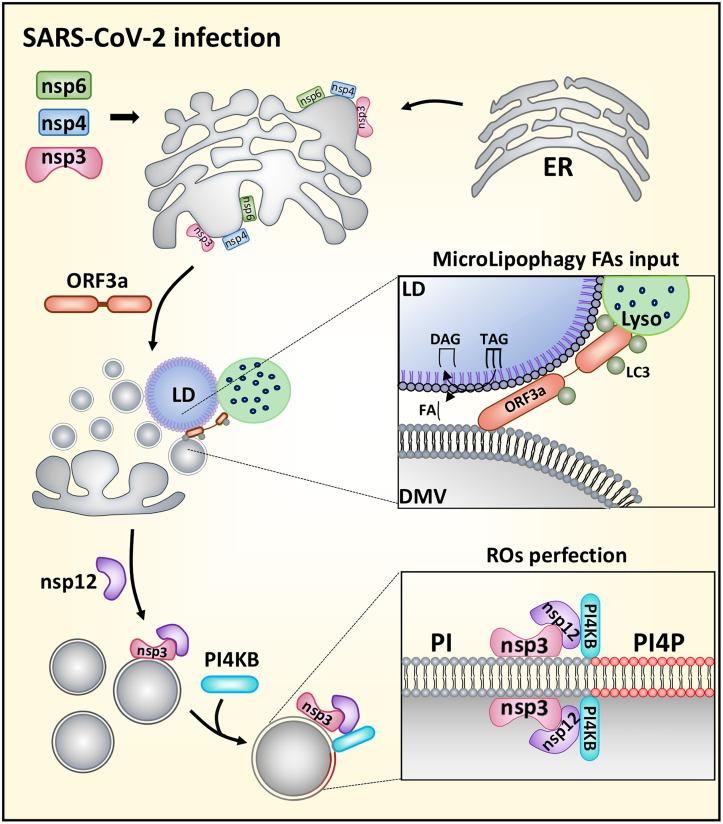
A model depicting microlipophagy induced by orf3a enhances biogenesis of SARS-CoV-2 replication organelle.

In this study, we demonstrate that the formation and modification of SARS-CoV-2 ROs involve the synergistic action of multiple viral and cellular proteins. As illustrated in our proposed model (Fig 8), orf3a collaborates with nsp3, nsp4, and nsp6 to facilitate the generation of ROs. By targeting LDs to ROs, orf3a creates novel membrane contact sites and initiates microlipophagy, thereby providing essential lipids for the expansion of the RO membrane. Following the formation of ROs, nsp3 indirectly recruits PI4KB through its association with nsp12, which is critical for synthesizing PI4P. This recruitment results in a lipid-enriched microenvironment characterized by high levels of PI4P, thereby promoting viral replication.

Our findings also suggest that the biogenesis of SARS-CoV-2 ROs shares similarities with that of other positive-strand RNA viruses, requiring multiple viral proteins and lipid metabolism processes. For instance, the enteroviral membrane protein 3A reorganizes the host secretory trafficking pathway to enhance the recruitment of PI4KB to the host membrane, generating a PI4P-rich environment conducive to the binding of soluble viral RNA-dependent RNA polymerase (RdRp) 3D^pol^ [12]. Moreover, certain picornaviruses exploit cellular lipid metabolism by targeting long-chain acyl-CoA synthetase activity, which augments the import of free fatty acids in infected cells and upregulates phospholipid synthesis, ultimately leading to the formation of ROs [42].

Although orf3a is classified as an accessory protein in coronaviruses, it plays a crucial role in various aspects of viral pathogenicity [43,44]. Both SARS-CoV and SARS-CoV-2 orf3a possess distinct functional domains associated with virulence, infectivity, and virion release [45]. In animal models of SARS-CoV infection, deletion of the orf3a gene significantly reduced viral replication [46]. Notably, substantial CD4^+^ and CD8^+^ T cell responses in individuals infected with SARS-CoV-2 were directed against orf3a expression [47]. Previous studies have demonstrated that SARS-CoV-2 orf3a inhibits autolysosome formation, thereby promoting lysosomal exocytosis-mediated viral egress, effectively blocking macroautophagy (Chen et al., 2021; Miao et al., 2021). However, to ensure a sufficient supply of FFAs for the formation and function of ROs, the virus employs orf3a to induce microautophagy. By marking and characterizing LDs, orf3a facilitates their accurate transport to lysosomes for degradation, thereby obtaining FFAs necessary for membrane expansion and modification. This viral strategy optimizes conditions for replication and budding. Orf3a exhibits diverse roles at different stages of the viral life cycle. Specifically, during the stage of viral genome replication, orf3a induces microlipophagy to provide FFAs essential for RO generation. Conversely, once sufficient RNA is available for translation and assembly, orf3a inhibits macroautophagy to promote lysosomal exocytosis-mediated viral egress. This dual functionality underscores the importance of orf3a in balancing the viral replication process and subsequent release of new virions.

Furthermore, evidence suggests that the accumulation of excess FFAs due to obesity can induce a chronic ER stress response, leading to the persistent activation of the unfolded protein response (UPR) pathway [48]. Consequently, we investigated whether SARS-CoV-2 infection induces ER stress or activates the UPR. Our findings revealed that the classical ER stress marker protein GRP78 was significantly elevated in Huh7 cells infected with SARS-CoV-2. This indicates that FFAs generated from virus-induced lipophagy may indeed trigger ER stress or the UPR. Thus, it is pertinent to explore whether and how FFAs produced as a result of lipophagy contribute to the induction of ER stress and the activation of the UPR.

It has been demonstrated that certain enterovirus infections promote the import of FFAs from extracellular media for phospholipid synthesis [42]. Additionally, evidence indicates that these FFAs, imported by enteroviruses, are not directly utilized for the biogenesis of viral ROs. Instead, they are converted into triacylglycerols (TGs) and stored in LDs. Subsequently, enteroviruses induce lipolysis of LDs, releasing FFAs for RO synthesis [15]. In our study, we found that orf3a is involved in inducing lipophagy, thereby providing a source of FFAs for RO synthesis. Notably, we also observed that expression of nsp1 increases the number of LDs (S2B Fig). This prompted us to hypothesize that SARS-CoV-2 infection leads to the import of FFAs from the media, where they are converted into TGs, facilitated by nsp1. These TGs are deposited in LDs, and orf3a can then induce lipophagy to release FFAs from LDs when necessary for the biogenesis of ROs. Further studies are warranted to explore the detailed mechanisms and host factors involved in this intricate process.

Our results indicate that ROs of SARS-CoV-2 form during the early stages of infection (Fig 1C). However, these vesicles lacked clear bilayer membrane characteristics, suggesting that the initial vesicle-like structures require subsequent processing and maturation. Given that the small GTPase Sar1 is a pivotal regulator of membrane bending, we aimed to investigate whether orf3a, nsp3, nsp4, or nsp6 can recruit Sar1 to facilitate the budding of viral ROs from the ER. As anticipated, we observed that Sar1 co-immunoprecipitated with nsp3 (S8A Fig). Additionally, following the knockdown of Sar1 expression using siRNAs, we found a significant decrease in ROs formation and SARS-CoV-2 infection levels (S8B and S8C Fig). These findings suggest that Sar1 may play a role in the generation of ROs, thereby promoting the replication of SARS-CoV-2. Future studies will be essential to elucidate the specific molecular mechanisms by which Sar1 contributes to the biogenesis of ROs.

In conclusion, our study elucidates a novel mechanism underlying the formation and modification of ROs in SARS-CoV-2. We demonstrate that orf3a cooperates with nsp3, nsp4, and nsp6 to regulate the biogenesis of ROs while simultaneously inducing microlipophagy to supply FFAs that are essential for the membrane composition of ROs. Furthermore, we establish that nsp3 collaborates with nsp12 to recruit PI4KB for the synthesis of PI4P, thereby creating a microenvironment enriched in PI4P that is conducive to viral replication. These results provide fascinating insights into the interplay between lipid metabolism and coronavirus replication. Importantly, the necessity of microlipophagy for SARS-CoV-2 replication suggests a potential therapeutic strategy that could interfere with the generation of coronavirus ROs by targeting and inhibiting the viral-induced microlipophagy process.

### Limitations and deficiencies

Firstly, while our findings indicate that orf3a induces microlipophagy and degrades LDs to synthesize FFAs, we have not yet examined whether the flux of FFAs occurs directly from LDs to ROs. Secondly, although we observed that the virus recruits PI4KB to ROs for PI4P production, the precise mechanism underlying PI4P synthesis remains unclear. Additionally, it remains to be determined whether the FFAs released via orf3a-induced microlipophagy serve as a direct source for PI4P synthesis. Furthermore, while our results suggest that ROs may derive from single-membrane vesicles (SMVs) budding from the ER at early stages of infection, the transition mechanism from SMVs to DMVs requires further investigation. These unresolved questions highlight critical areas for future research to enhance our understanding of the molecular dynamics involved in the biogenesis of ROs and their role in viral replication.

## STAR★Methods

### Experimental model and subject details

Experimental cells (HeLa cells, A549 cells, HEK293T cells) were obtained from ACTCC, experimental cells (huh7 cells, Vero E6 cells) were obtained from Huazhong Agricultural University, and cells were cultured in Dulbecco’s modified Eagle’s medium (DMEM; GIBCO) supplemented with 10% fetal bovine serum (FBS; Gibco) and 100 U/ml penicillin/streptomycin (Gibco) at 37°C with 5% CO_2_.

### Method details

#### Antibodies and reagents.

Mouse anti-Tom20 monoclonal antibody (Ab) was purchased from BD Biosciences. Rabbit anti-Calnexin polyclonal Ab was purchased from Sigma-Aldrich. Rabbit anti-PI4KA polyclonal Ab was purchased from ABclonal Technology. Rabbit anti-PI4KB and anti-PI4K2B polyclonal Abs were purchased from Abcam. Rabbit anti-PI42A polyclonal Ab was purchased from Cell Signaling Technology. Mouse anti-PI4P monoclonal Ab was purchased from Echelon Biosciences. Rabbit anti-Flag polyclonal Ab and mouse anti-HA monoclonal Ab were purchased from Sigma-Aldrich. Mouse anti-Myc and anti-GAPDH monoclonal Abs were purchased from Santa Cruz Biotechnology, Inc. Anti-strep-tag II polyclonal Ab was from Abcam. Anti-strep-tag II monoclonal Ab was from Abclonal. Anti-SARS-CoV-2-N polyclonal Ab was from Sino Biological. Anti-dsRNA monoclonal Ab was from SCICONS. Mouse anti-LAMP1 and anti-LAMP2 monoclonal Abs were from Santa Cruz Biotechnology, Inc. Goat anti-rabbit IgG Rhodamine and goat anti-mouse IgG Fluorescein were from Thermo. DAPI was from Sigma-Aldrich.

#### Infection and transfection.

For infection, VeroE6 or huh7 cells were infected with DMEM containing viruses with a multiplicity of infection (MOI) as indicated in the figure legends. 2 h later, the medium was replaced with fresh DMEM with 10% FBS. All experiments involving live viruses were performed in a biosafety level 3 (BSL3) facility at Huazhong Agricultural University in accordance with the institutional biosafety manual. For transfection, plasmids were transfected using Lipofectamine 2000 (Invitrogen) according to the manufacturer’s instructions, and cells were harvested or further treated as indicated.

#### SDS-PAGE and western blot.

Cells were harvested and lysed with lysis buffer (150 nM NaCl, 50 nM Tris-HCL [PH 7.4], 1% Triton X-100, 1 mM EDTA [PH 8.0], 0.1% SDS with a protease inhibitor cocktail) for 30 min at 4°C. The supernatants were collected by centrifugation at 12000 g for 25 min at 4°C. Protein concentrations were determined based on the Bradford method. Equal amounts of proteins were separated by 10% sodium dodecyl sulfate-polyacrylamide gel electrophoresis (SDS-PAGE) and transferred onto a nitrocellulose membrane (GE Healthcare). After blocking with 5% non-fat milk dissolved in PBST (phosphate-buffered saline with 0.1% Tween 20), the membrane was incubated with the primary Abs, followed by horseradish peroxidase-conjugated goat anti-rabbit or anti-mouse IgG (Thermo Fisher Scientific). The proteins were detected on a Fujifilm LAS-4000 imaging system using an immobilon western HP substrate (Millipore). The primary antibodies used were as follows: mouse anti-SARS-CoV-2-N (1:10000), mouse anti-GAPDH (1:10000), mouse anti-HA (1:10000), mouse anti-Flag (1:5000), mouse anti-Myc (1:5000), rabbit anti-LC3B (1:2000), rabbit anti-p62 (1:2000), mouse anti-Tom20 (1:5000), rabbit anti-calnexin (1:5000), mouse anti-strep-tag II (1:5000), rabbit anti-PI4KA (1:1000), rabbit anti-PI42A (1:500), rabbit anti-PI4KB (1:5000), rabbit anti-PI42B (1:100), HRP-conjugated goat anti-mouse immunoglobulin (IgG) (1:5000) and goat anti-rabbit IgG (1:5000) were used as secondary antibodies.

### Immunofluorescence analysis

In a third-level biological safety protection laboratory, Huh7 cells and VeroE6 were cultured in 24-well plates with coverslips and transfected with the indicated plasmids or infected with SARS-CoV-2. Cells were washed with phosphate-buffered saline (PBS) three times (5 min each time) and fixed with 4% (wt/vol) paraformaldehyde/PBS for 30 min at room temperature. Then Cells were washed with PBS three times (5 min each time) and incubated with 50 μg/ml digitonin diluted with PBS for 5 min and washed with PBS three times (5 min each time). Next, cells were blocked with 3% (wt/vol) bovine serum albumin (BSA)/PBS at room temperature for 30 min and incubated with primary Abs diluted in 1% (wt/vol) BSA/PBS for 1 h at 4°C. Cells were washed with PBS three times (once in 5 min) and incubated with secondary antibodies diluted in 1% (wt/vol) BSA/PBS for 1 h. After being stained with DAPI (Sigma) for 5 min, cells were observed with a confocal microscope.

The primary antibodies used were as follows: rabbit anti-strep-tag II (1:2000), mouse anti-HA (1:1000), rabbit anti-SARS-CoV-2-N (1:5000), mouse anti-PI4P (1:500), rabbit anti-PI4KA (1:100), rabbit anti-PI4KB (1:500), rabbit anti-PI4K2A (1:100), rabbit anti-PI4K2B (1:100), rabbit anti-Flag (1:1000), mouse anti-Myc (1:1000), mouse anti-J2 (1:500). All images were captured through Leica confocal microscopy unless otherwise marked.

### COIP using cell lysates

HEK293T cells were transfected with the indicated plasmids for 36 h. Cells were harvested and lysed with 400 μl lysis buffer (150 mM NaCl, 50 mM Tris-HCl [pH,7.4], 1%TritonX-100, 1 mM EDTA [pH,8.0], and 0.1% SDS and protease inhibitor cocktail) for 30 min on ice. The supernatants were collected via centrifugation at 12000 g for 30 min at 4°C. Next, 50 μl supernatants were added with loading buffer and boiled at 100°C for 10 min. Additionally, 350 μl supernatants were precleared by adding 20 μl protein G Sepharose 4 Fast Flow beads at 4°C with rotation for 2 h. Supernatants were collected via centrifugation at 5000 g 4°C for 2 min. The indicated primary Abs and 30 μl beads were added and incubated at 4°C with rotation overnight. Beads were collected via centrifugation at 5000 g for 2 min and washed with lysis buffer three times (2 min each time). Next, beads were boiled with loading buffer at 100°C for 10 min, and the proteins were analyzed via WB.

### Transmission electron microscopy

HeLa cells were transfected with the indicated plasmids or huh7 and veroE6 cells were infected with SARS-CoV-2, and the cells were fixed with fixative liquid (3% paraformaldehyde and 1.5% glutaraldehyde in 0.1M sodium phosphate buffer [pH,7.4]) for 1 h at room temperature, harvested, and subjected to gradient centrifugation (1000 g, 5 min; 3000 g, 5 min; 6000 g, 5 min; 12000 g, 5 min) at 4°C, post-fixed with 1% osmium tetroxide for 1 h at 4°C under dark conditions, incubated with 2% uranyl acetate overnight, dehydrated in increasing concentrations of ethanol (50%, 75%, 95%, and 100%), and processed for embedding in epoxy resin. Ultrathin (~ 70 nm) sections were collected on uncoated 200-mesh copper grids, stained with uranyl acetate and lead citrate, and evaluated via transmission electron microscopy (JEOL, JEM-1400 plus) operating at 100 kV.

### Lipid droplet purification

Cells from ten 150 mm culture plates were collected by centrifugation and washed with PBS. The pellet was resuspended in 2 mL of Buffer A (20 mM Tricine, 250 mM sucrose, 0.2 mM PMSF, pH 7.8) and incubated on ice for 20 min. The suspension was then homogenized with a 5 mL tissue grinder using 20 strokes on ice. The homogenate was centrifuged at 3,000 × g for 10 min at 4°C to collect the supernatant. The supernatant was transferred to a clear SW40 centrifuge tube, adjusted to 10 mL with Buffer A, and carefully overlaid with 4 mL of Buffer B (20 mM HEPES, 100 mM KCl, 2 mM MgCl₂, pH 7.4) to form a discontinuous gradient. Centrifugation was performed at 38,000 × g for 1 h at 4°C with slow acceleration and deceleration. The lipid droplet fraction was collected from the top of the gradient, washed five times with Buffer B, and finally resuspended in SDS protein loading buffer. The sample was boiled at 100°C for 10 min and subjected to Western blot analysis.

### Plasmids and shRNA Oligonucleotides

Double-strand oligonucleotides corresponding to the target sequences were cloned into pLKO.1. The target sequences for PI4KA were as follows: GCGTCTCATCACATGGTACAA. The target sequences for PI4KB were as follows: GCAAGAAACACGAAGGATCAT. The target sequences for PI4K2A were as follows: CAATGACAACTGGCTGATTAA. The target sequences of PI4K2B were as follows: GCTGCAATTGATAATGGTCTA.

### Free fatty quantitation

HeLa cells (1 × 10^7^) were transfected with empty plasmid or orf3a-strep for 36h, cells were harvested and homogenized in 200 μl of a 1% (w/v) Triton X-100 in chloroform solution. The samples were centrifuged at 13,000 g for 10 min to remove insoluble material. The organic phases (low phase) was collected and air dried at 50°C to remove chloroform, and then was vacuum dried for 30 min to remove trace chloroform. The dried lipids were dissolved in 200 μl of Fatty Acid Assay buffer by vortexing extensively for 5 min, and were added 2 μl of Acyl-CoA synthetase (ACS) reagent to each sample and standard well, incubated for 30 min at 37°C. The master reaction mix was set up according to the protocol, and each of the wells were added 50 μl of the master reaction mix using a horizontal shaker or pipetting, the reaction was incubated for 30 min at 37°C, and the absorbance was measured at 570 nm (A570) using colorimetric assays.

### Quantification and statistical analysis

Statistical parameters, including the definition and exact values of n, distribution, and deviation, are reported in the figure legends. Data are expressed as mean ± standard deviation (SD). The significance of the variability between different groups was determined by two-way analyses of variance using GraphPad Prism software (version 5.0). A *P* value of <0.05 was considered statistically significant, and a *P*-value of >0.05 was considered statistically non-significant.

## Supporting information

S1 Fig Recruitment of LDs to ROs generates membrane contact sites and facilitates LDs degradation in SARS-CoV-2 infected cells, related to Fig 1. (A) Low magnification transmission electron micrograph of SARS-CoV-2 infected huh7 cells with (MOI = 2) for 0 h, 12 h, and 24 h, and analyzed to access the structure of the ER and ROs. Red arrowheads indicated the ER structures, and yellow arrowheads indicated the ROs. M, Mitochondria. Scale bars, 1μm. (B) Quantification of the number of ROs per cell after SARS-CoV-2 infection at 0 h, 12 h, 24 h. (C-D) Low magnification transmission electron micrograph of SARS-CoV-2 infected huh7 cells with (MOI = 2) or veroE6 cells (MOI = 0.05) at 24 h, and analyzed to access the structure of the multi-membrane vesicles (MMVs). Blue arrowheads indicated the MMVs. Scale bars, 500nm. (E-F) Huh7 cells were infected with SARS-COV-2 (MOI = 2) for 24 h and then analyzed to detect the number of LDs, SARS-CoV-2 was labeled with anti-dsRNA antibody (red), and LDs was labeled with Bodipy493 (green), and quantification of the number of LDs per cell. The plus sign marks the cells which were infected with SARS-CoV-2. Error bars, mean ± SD of three experiments (n = 3). Student t-test; ^★^*P* < 0.05; ^★★^*P* < 0.01; ^★★★^*P* < 0.001.
(TIF)

S2 Fig Orf3a is responsible for recruiting LDs to ROs, related to Fig 2. (A) SARS-CoV-2 structural proteins were not responsible for LDs recruitment. HeLa cells were transfected with each structural protein for 24 h and analyzed the distribution of LDs. (B) SARS-CoV-2 nonstructural proteins were not responsible for LDs recruitment. HeLa cells were transfected with each nonstructural protein for 24 h and analyzed the distribution of LDs. (C) SARS-CoV-2 accessory proteins were not responsible for LDs recruitment. HeLa cells were transfected with each accessory protein for 24 h and analyzed the distribution of LDs. (D) A549 cells were transfected orf3a-strep for 24 h to confirm the distribution relationship between LDs and orf3a. orf3a-strep was labeled with anti-strep antibodies (red), LDs were labeled with Bodipy493 (green). (E-F) HeLa cells were transfected Flag-TIP47 or Flag-TIP47 and orf3a-strep for 24 h, analyzed the distribution of Flag-TIP47, and quantification of the mean intensity of TIP47 on LDs. orf3a-strep was labeled with anti-strep antibodies (red), Flag-TIP47 was labeled with anti-Flag antibodies (green). Error bars, mean ± SD of three experiments (n = 3). Student t-test; ^★^*P* < 0.05; ^★★^*P* < 0.01; ^★★★^*P* < 0.001. Scale bars, 10µm.
(TIF)

S3 Fig Orf3a-dependent microlipophagy enhances RO biogenesis through LD recruitment, related to Fig 3. (A-B) HeLa cells were transfected with empty plasmid or strep-tagged orf3a for 24 h and then analyzed to detect the localization and protein level of PLIN3. (C-D) A549 cells were transfected with empty plasmid or strep-tagged orf3a for 24 h and then analyzed to detect the localization of orf3a and lysosome marker LAMP1 or LAMP2. (E-F) A549 cells were transfected with empty plasmid or strep-tagged orf3a for 24 h and then analyzed to detect the localization of LDs and lysosome marker LAMP1 or LAMP2. (G) A549 cells were transfected with GFP-LC3 or GFP-LC3 and strep-tagged orf3a for 24h and then analyzed to detect the localization of LAMP1 and GFP-LC3. Scale bars, 10µm.
(TIF)

S4 Fig Orf3a-dependent microlipophagy enhances RO biogenesis through LD recruitment related to Fig 5. (A-D) HeLa cells were transfected with indicated plasmids for 30 h and analyzed via electron microscopy to assess the ultrastructure of the vesicles. (A) ER structures were rich in HeLa cells transfected with nsp3. (B) Single membrane vesicles (SMVs) were found in HeLa cells transfected with nsp4. (C) Maze-like bodies were found in HeLa cells transfected with nsp4. (D) SMVs were found in HeLa cells transfected with nsp6. Scale bars, 1µm. (F-G) HeLa cells were transfected with the indicated plasmids for 24 h and then analyzed to detect the distribution of ER marker Calnexin. Scale bars, 10µm.
(TIF)

S5 Fig PI4KB is recruited to SARS-CoV-2 ROs to generate PI4P, related to Fig 6. (A-D) Huh7 cells were transfected with shRNA targeting PI4KA, PI4KB, PI4K2A, PI4K2B respectively, and infected with SARS-CoV-2 (MOI = 2) for 24 h, and then analyzed the N protein via WB.
(TIF)

S6 Figs PI4KB is recruited to SARS-CoV-2 ROs via its interaction with nsp3, related to Fig 7. Structural proteins, accessory proteins, or nonstructural proteins other than nsp12 and nsp13 did not interact with PI4Kase. HEK293T cells were transfected with the indicated plasmids for 36 h. Cell lysates were subjected to IP and analyzed via WB.
(TIF)

S7 Figs PI4KB is recruited to SARS-CoV-2 ROs via its interaction with nsp3, related to Fig 7. Structural proteins, accessory proteins, or nonstructural proteins other than nsp12 and nsp13 did not interact with PI4Kase. HEK293T cells were transfected with the indicated plasmids for 36 h. Cell lysates were subjected to IP and analyzed via WB.
(TIF)

S8 Fig Sar1 is required for ROs formation and SARS-CoV-2 replication. (A) HEK293T cells were transfected with indicated plasmids, then analyzed via WB. (B) WT Hela or Sar1 KO cells were transfected with nsp3/nsp4/nsp6/orf3a, and analyzed the ROs via TEM. (C) WT Hela or Sar1 KO cells were infected with SARS-CoV-2, and analyzed via WB.
(TIF)
