# Supplementary figures and images for: Reprogramming of lipid metabolism by ORF3a-induced microlipophagy enhances biogenesis of SARS-CoV-2 replication organelle
Source: PLoS Pathog. 2025 Nov 21;21(11):e1013676. doi: 10.1371/journal.ppat.1013676 (PMC12671888; doi:10.1371/journal.ppat.1013676)

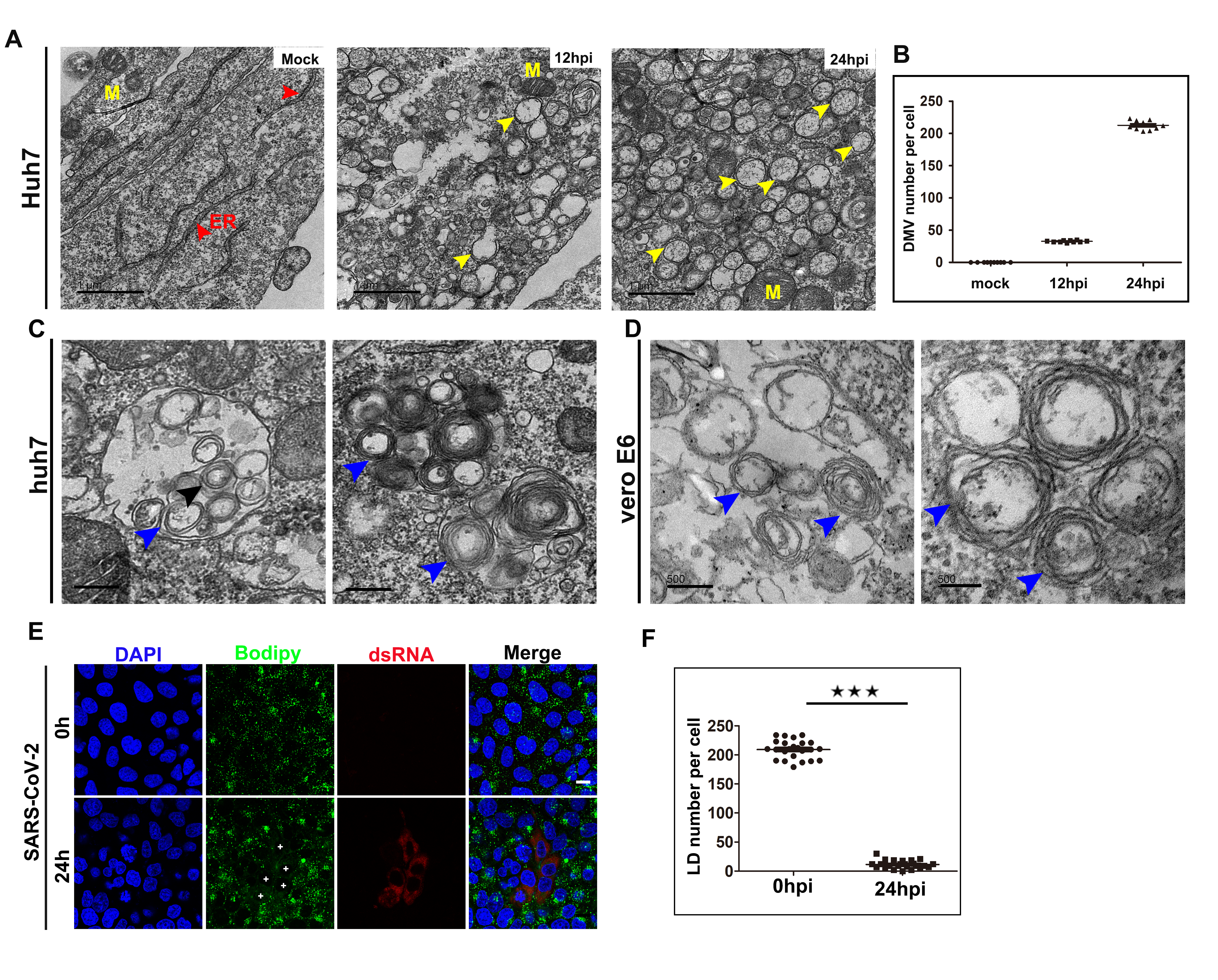

Supplement: S1 Fig — (A) Low magnification transmission electron micrograph of SARS-CoV-2 infected huh7 cells with (MOI = 2) for 0 h, 12 h, and 24 h, and analyzed to access the structure of the ER and ROs. Red arrowheads indicated the ER structures, and yellow arrowheads indicated the ROs. M, Mitochondria. Scale bars, 1μm. (B) Quantification of the number of ROs per cell after SARS-CoV-2 infection at 0 h, 12 h, 24 h. (C-D) Low magnification transmission electron micrograph of SARS-CoV-2 infected huh7 cells with (MOI = 2) or veroE6 cells (MOI = 0.05) at 24 h, and analyzed to access the structure of the multi-membrane vesicles (MMVs). Blue arrowheads indicated the MMVs. Scale bars, 500nm. (E-F) Huh7 cells were infected with SARS-COV-2 (MOI = 2) for 24 h and then analyzed to detect the number of LDs, SARS-CoV-2 was labeled with anti-dsRNA antibody (red), and LDs was labeled with Bodipy493 (green), and quantification of the number of LDs per cell. The plus sign marks the cells which were infected with SARS-CoV-2. Error bars, mean ± SD of three experiments (n = 3). Student t-test; ★P < 0.05; ★★P < 0.01; ★★★P < 0.001. (TIF) [file ppat.1013676.s001.tif]

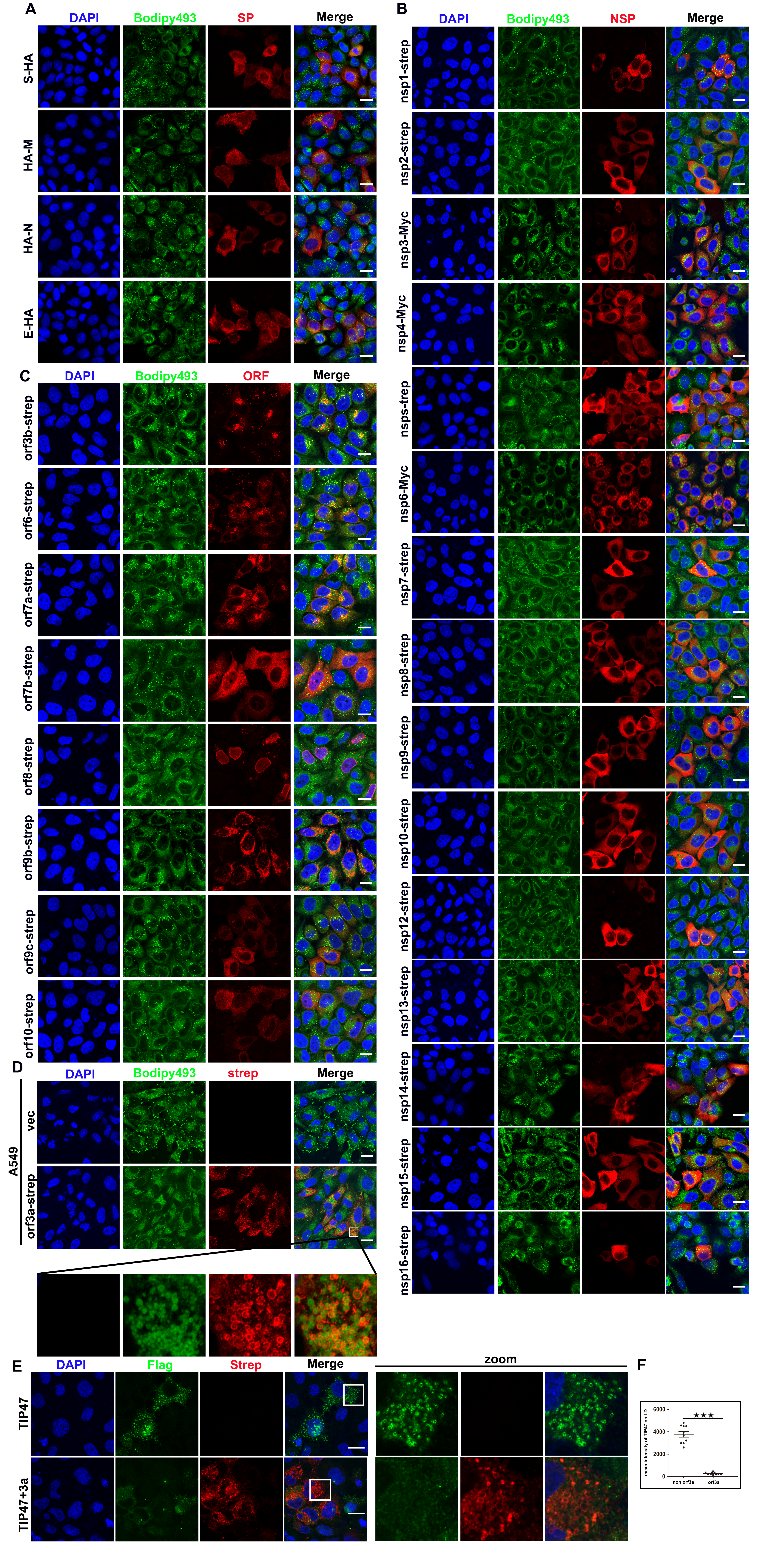

Supplement: S2 Fig — (A) SARS-CoV-2 structural proteins were not responsible for LDs recruitment. HeLa cells were transfected with each structural protein for 24 h and analyzed the distribution of LDs. (B) SARS-CoV-2 nonstructural proteins were not responsible for LDs recruitment. HeLa cells were transfected with each nonstructural protein for 24 h and analyzed the distribution of LDs. (C) SARS-CoV-2 accessory proteins were not responsible for LDs recruitment. HeLa cells were transfected with each accessory protein for 24 h and analyzed the distribution of LDs. (D) A549 cells were transfected orf3a-strep for 24 h to confirm the distribution relationship between LDs and orf3a. orf3a-strep was labeled with anti-strep antibodies (red), LDs were labeled with Bodipy493 (green). (E-F) HeLa cells were transfected Flag-TIP47 or Flag-TIP47 and orf3a-strep for 24 h, analyzed the distribution of Flag-TIP47, and quantification of the mean intensity of TIP47 on LDs. orf3a-strep was labeled with anti-strep antibodies (red), Flag-TIP47 was labeled with anti-Flag antibodies (green). Error bars, mean ± SD of three experiments (n = 3). Student t-test; ★P < 0.05; ★★P < 0.01; ★★★P < 0.001. Scale bars, 10µm. (TIF) [file ppat.1013676.s002.tif]

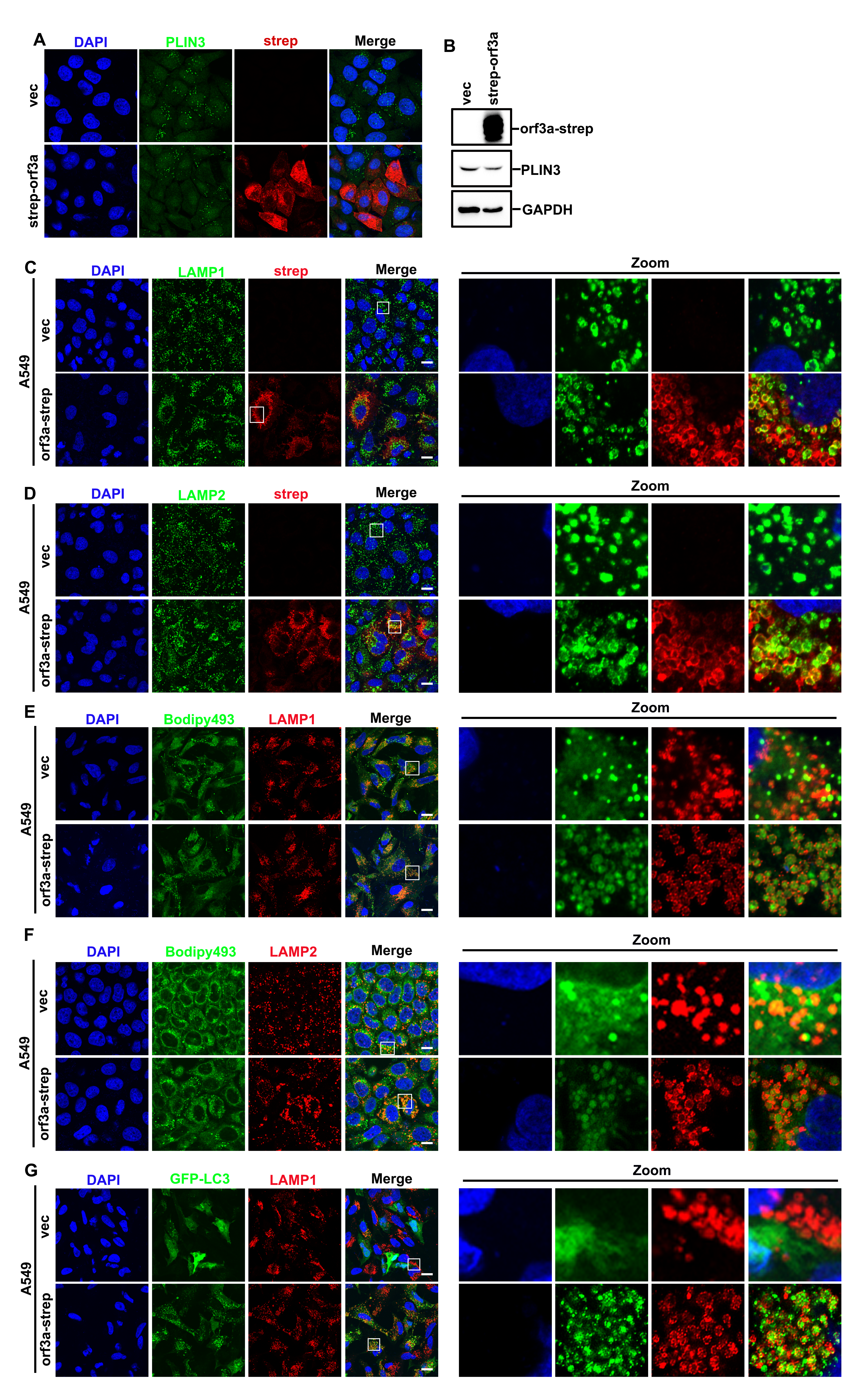

Supplement: S3 Fig — (A-B) HeLa cells were transfected with empty plasmid or strep-tagged orf3a for 24 h and then analyzed to detect the localization and protein level of PLIN3. (C-D) A549 cells were transfected with empty plasmid or strep-tagged orf3a for 24 h and then analyzed to detect the localization of orf3a and lysosome marker LAMP1 or LAMP2. (E-F) A549 cells were transfected with empty plasmid or strep-tagged orf3a for 24 h and then analyzed to detect the localization of LDs and lysosome marker LAMP1 or LAMP2. (G) A549 cells were transfected with GFP-LC3 or GFP-LC3 and strep-tagged orf3a for 24h and then analyzed to detect the localization of LAMP1 and GFP-LC3. Scale bars, 10µm. (TIF) [file ppat.1013676.s003.tif]

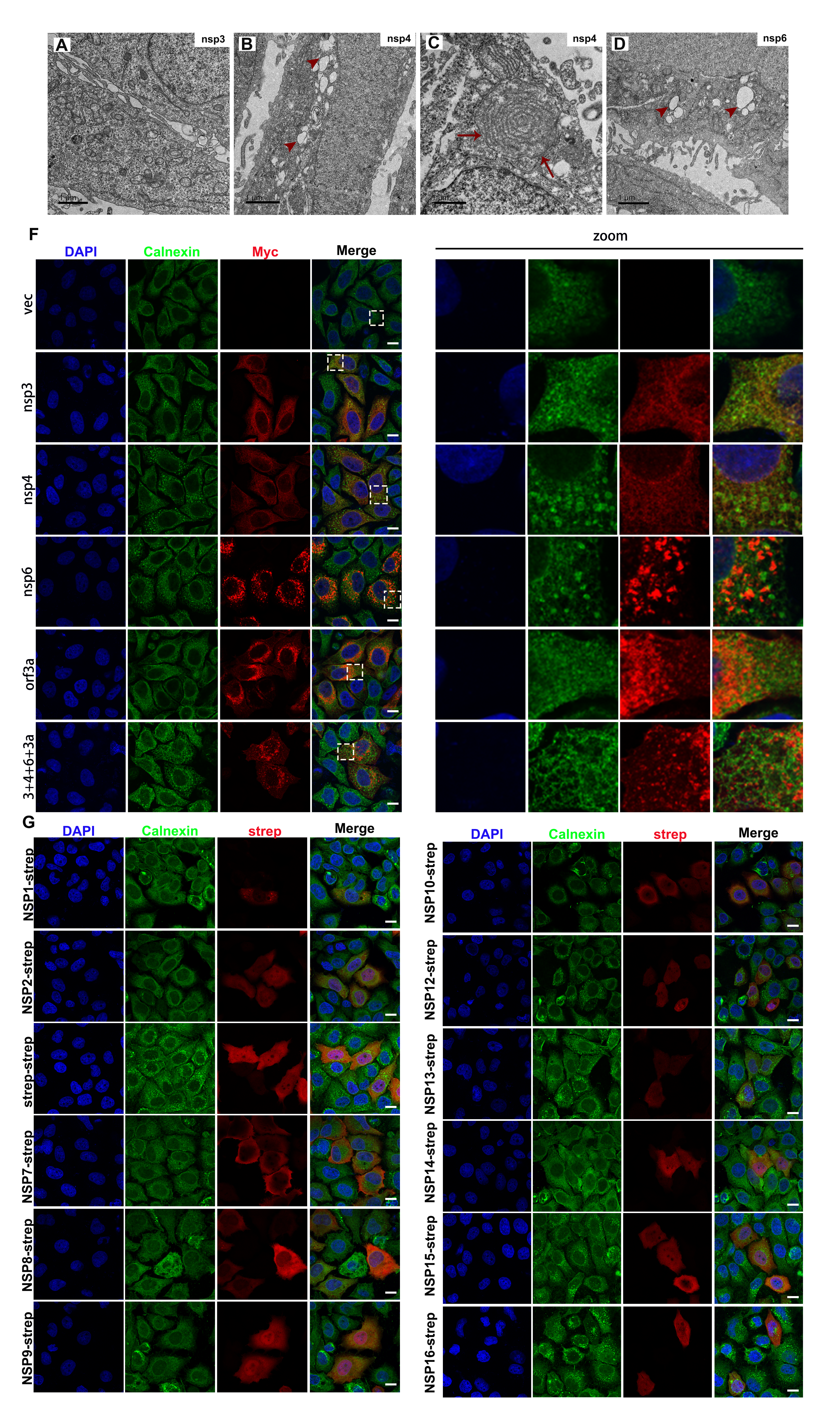

Supplement: S4 Fig — (A-D) HeLa cells were transfected with indicated plasmids for 30 h and analyzed via electron microscopy to assess the ultrastructure of the vesicles. (A) ER structures were rich in HeLa cells transfected with nsp3. (B) Single membrane vesicles (SMVs) were found in HeLa cells transfected with nsp4. (C) Maze-like bodies were found in HeLa cells transfected with nsp4. (D) SMVs were found in HeLa cells transfected with nsp6. Scale bars, 1µm. (F-G) HeLa cells were transfected with the indicated plasmids for 24 h and then analyzed to detect the distribution of ER marker Calnexin. Scale bars, 10µm. (TIF) [file ppat.1013676.s004.tif]

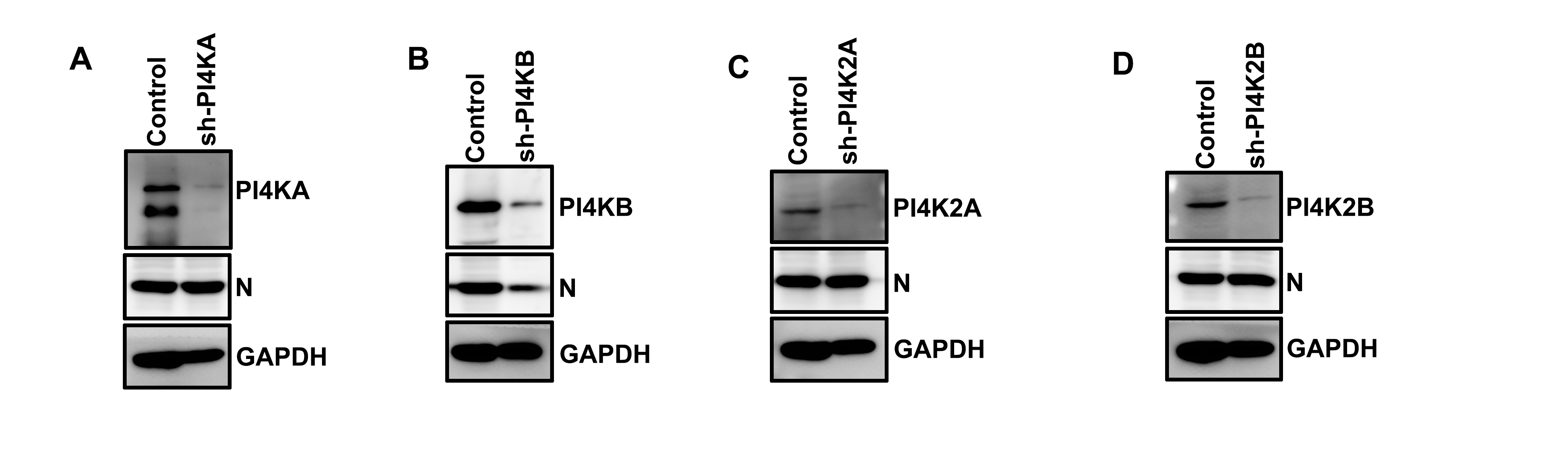

Supplement: S5 Fig — (A-D) Huh7 cells were transfected with shRNA targeting PI4KA, PI4KB, PI4K2A, PI4K2B respectively, and infected with SARS-CoV-2 (MOI = 2) for 24 h, and then analyzed the N protein via WB. (TIF) [file ppat.1013676.s005.tif]

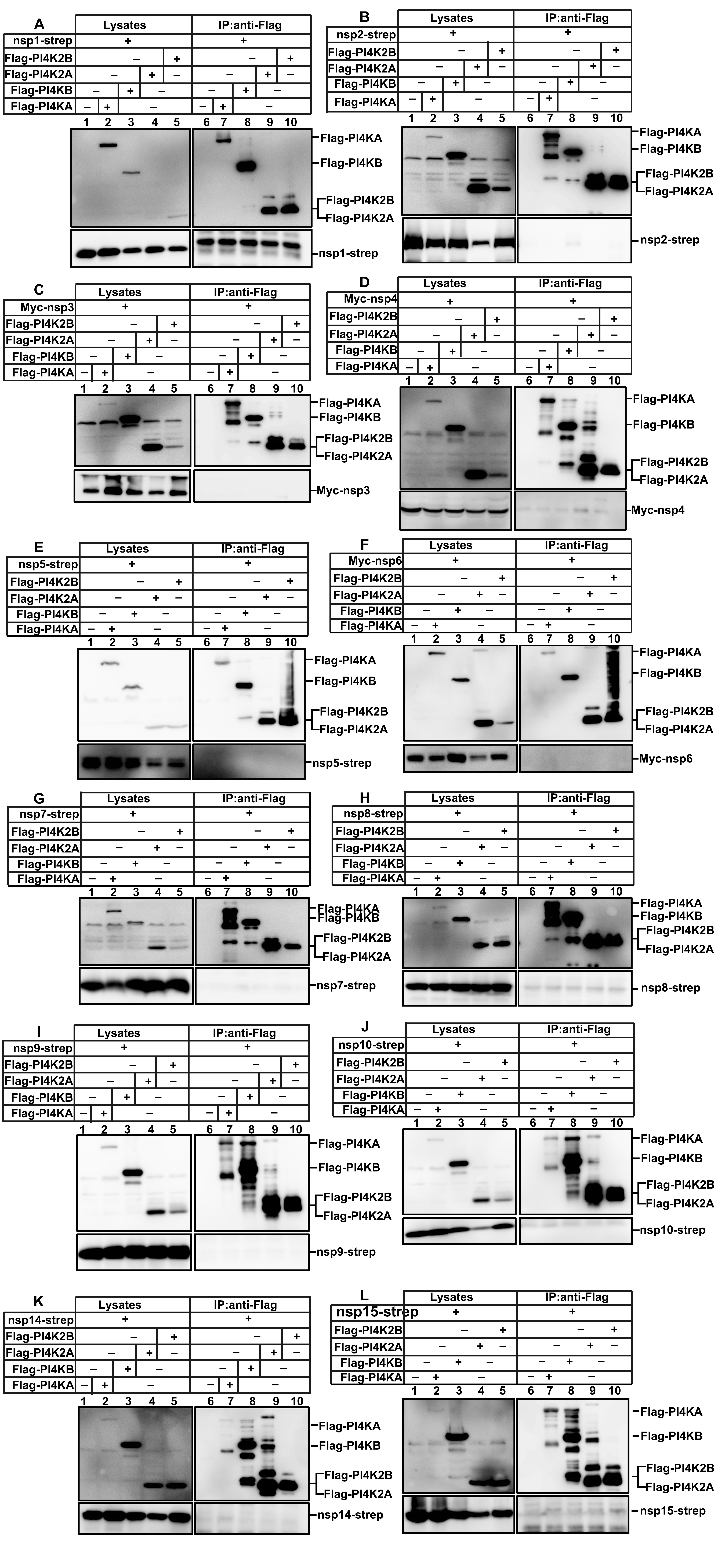

Supplement: S6 Figs — Structural proteins, accessory proteins, or nonstructural proteins other than nsp12 and nsp13 did not interact with PI4Kase. HEK293T cells were transfected with the indicated plasmids for 36 h. Cell lysates were subjected to IP and analyzed via WB. (TIF) [file ppat.1013676.s006.tif]

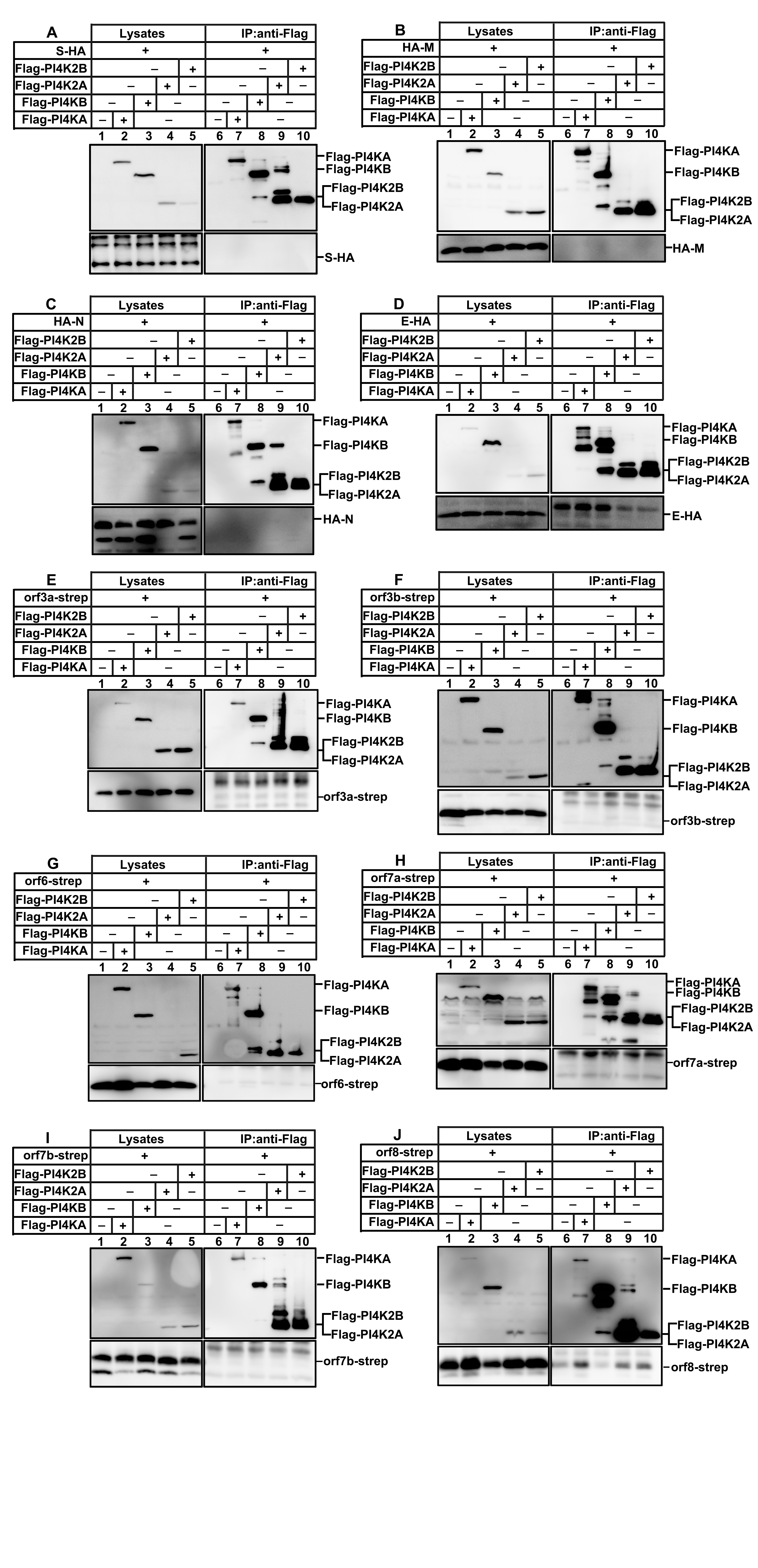

Supplement: S7 Figs — Structural proteins, accessory proteins, or nonstructural proteins other than nsp12 and nsp13 did not interact with PI4Kase. HEK293T cells were transfected with the indicated plasmids for 36 h. Cell lysates were subjected to IP and analyzed via WB. (TIF) [file ppat.1013676.s007.tif]

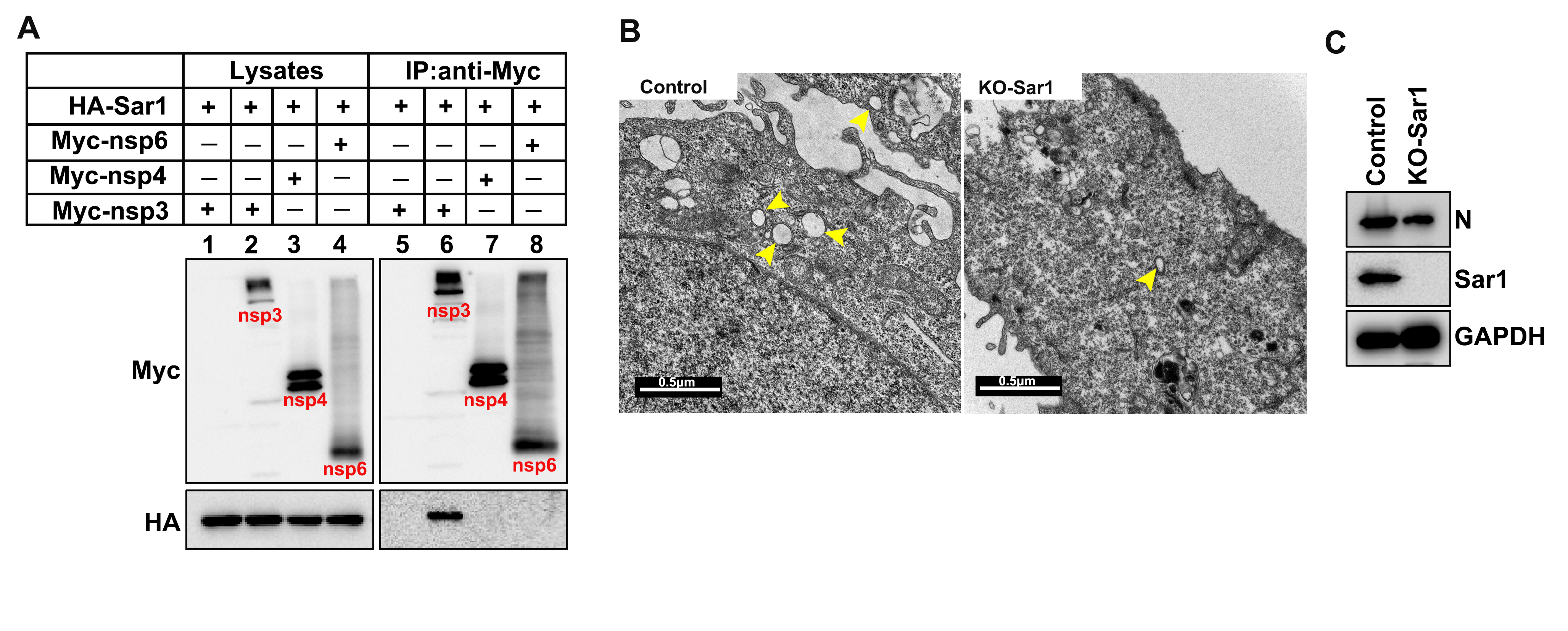

Supplement: S8 Fig — (A) HEK293T cells were transfected with indicated plasmids, then analyzed via WB. (B) WT Hela or Sar1 KO cells were transfected with nsp3/nsp4/nsp6/orf3a, and analyzed the ROs via TEM. (C) WT Hela or Sar1 KO cells were infected with SARS-CoV-2, and analyzed via WB. (TIF) [file ppat.1013676.s008.tif]
